# Co-Encapsulation of Multiple Antineoplastic Agents in Liposomes by Exploring Microfluidics

**DOI:** 10.3390/ijms26083820

**Published:** 2025-04-17

**Authors:** Sajid Asghar, Radu Iliescu, Rares-Ionut Stiufiuc, Brindusa Dragoi

**Affiliations:** 1 Nanotechnology Laboratory, TRANSCEND Department, Regional Institute of Oncology, 2-4 General Henri Mathias Berthelot, 700483 Iași, Romania; sajuhappa@gmail.com; 2 Department of Pharmaceutics, Faculty of Pharmaceutical Sciences, Government College University, Faisalabad 38000, Pakistan; 3 Proteomics Laboratory, TRANSCEND Research Center, Regional Institute of Oncology, 2-4 General Henri Mathias Berthelot Street, 700483 Iași, Romania; 4 Department of Pharmacology, Faculty of Medicine, Grigore T. Popa University of Medicine and Pharmacy, 16 University Street, 700115 Iași, Romania; 5 Department of NanoSciences, MEDFUTURE—Institute for Biomedical Research, “Iuliu Hatieganu” University of Medicine and Pharmacy, 4-6 Pasteur Street, 400349 Cluj-Napoca, Romania; 6 Faculty of Chemistry, Alexandru Ioan Cuza University of Iași, 11 Bd. Carol I, 700506 Iași, Romania

**Keywords:** multiple drug therapy, liposomes, microfluidics, cancer, drug delivery

## Abstract

The inherent complexity of cancer proliferation and malignancy cannot be addressed by the conventional approach of relying on high doses of a single powerful anticancer agent, which is associated with poor efficacy, higher toxicity, and the development of drug resistance. Multiple drug therapy (MDT) rationally designed to target tumor heterogeneity, block alternative survival pathways, modulate the tumor microenvironment, and reduce toxicities would be a viable solution against cancer. Liposomes are the most suitable carrier for anticancer MDT due to their ability to encapsulate both hydrophilic and hydrophobic agents, biocompatibility, and controlled release properties; however, an adequate manufacturing method is important for effective co-encapsulation. Microfluidics involves the manipulation of fluids at the microscale for the controlled synthesis of liposomes with desirable properties. This work critically reviews the use of microfluidics for the synthesis of anticancer MDT liposomes. MDT success not only relies on the identification of synergistic dose combinations of the anticancer modalities but also warrants the loading of multiple therapeutic entities within liposomes in optimal ratios, the protection of the drugs by the nanocarrier during systemic circulation, and the synchronous release at the target site in the same pattern as confirmed in preliminary efficacy studies. Prospects have been identified for the bench-to-bedside translation of anticancer MDT liposomes using microfluidics.

## 1. Introduction

Due to the complexity of diseases, targeting multiple molecular or cellular pathways may be a better approach to treatment. Multiple drug therapy (MDT), or combination therapy, involves using two or more drugs together to treat a disease [1]. Due to its potential to increase patient compliance, decrease the emergence of medication resistance, and improve treatment results, this strategy has attracted a lot of interest recently. The main reason for combining multiple drugs is to maximize their effectiveness without increasing the risk of side effects. This strategy is crucial for the treatment of cancer [2,3], heart diseases [4,5], metabolic disorders [6,7], renal malfunctions [8], neurological diseases [9,10], inflammatory diseases [11,12], and fatal infections like malaria [13], acquired immunodeficiency syndrome (AIDS) [14,15], tuberculosis [16,17], and leprosy [18]. Using a single medication too often can quickly result in drug resistance, whereas using a drug combination lowers the chance of resistance considerably and shortens the course of treatment, which improves costs and patient outcomes [19].

## 2. Drug Combinations in Cancer Treatment

Cancer, a substantial global health concern, is characterized by the loss of the ability of the body to control the proliferation and growth of abnormally transformed cells, which can also affect normal cells and spread throughout the body [20]. Conventional cancer treatments, such as radiation, surgery, and single-agent chemotherapy, may end up doing more harm due to the serious side effects compromising the patient’s quality of life and leading to drug resistance [21]. Combination therapy is designed to target numerous pathways or various cellular subpopulations at once and to avoid or delay resistance. This strategy can lower the risk of treatment failure brought on by resistant clones. Moreover, a single anticancer agent used in high quantities can be extremely toxic, which reduces the treatment’s effectiveness. Hence, combining several drugs at lower doses allows clinicians to decrease the incidence and severity of adverse effects and to improve the success rate of cancer therapy [22]. Table 1 displays the established drug combinations that are used in clinical practice for the treatment of different types of cancer.

Synergy in drug combinations occurs when their combined effect is greater than the sum of their individual effects [24]. To achieve synergy, drug combinations can be made to target different stages of the cell cycle, thus maximizing the likelihood of killing cancer cells, blocking alternative or sequential pathways necessary for cancer cell survival, or changing the tumor microenvironment.

### 2.1. Targeting Tumor Heterogeneity

Tumor heterogeneity contributes to cancer treatment resistance and occurs in all cancer types [25]. Multiple drug therapy seems a convenient way to suppress the clonal expansion of different types of cell populations within the tumor by targeting diverse pathways or cell cycle stages [26]. Researchers showed that Camptothecin (a topoisomerase inhibitor and an irinotecan analogue), when combined with CHEK1 inhibitors, was more effective against double-mutant colon cancer cell lines than the monotherapy [27].

### 2.2. Blocking Alternative Survival Pathways

Cancer cells exploit genetic damage to evade cell death. They can even exploit it for survival when monotherapy is employed, as these cells adapt rapidly and take advantage of compensatory survival mechanisms. For instance, cancer cells have been reported to upregulate the PI3K/AKT/mTOR pathway for their survival and proliferation when targeted with MAPK/ERK pathway inhibitors [28]. The resistance of the epithelial ovarian cancer cells against cisplatin was found to be overcome by concomitant treatment with BEZ235, a dual PI3K/mTOR inhibitor [29].

### 2.3. Modulation of Tumor Microenvironment

The tumor microenvironment plays a key role in cancer growth, spread, and drug resistance. The lack of success of anti-tumor therapy is mostly attributed to the complex nature of this microenvironment and its low level of understanding [30]. Targeting different aspects of the tumor microenvironment could diminish the survival chances of cancerous cells against therapy. Anti-angiogenesis therapy combined with chemotherapy has shown that the decreased blood supply to the tumor tissues can change the tumor microenvironment by starving the cancer cells, thus increasing the efficacy of co-administered chemotherapy [31].

### 2.4. Targeting Tumor-Associated Inflammatory Microenvironment

Inflammation is an important cancer hallmark [32] that affects the development, progression, and malignancy of various cancers [33]. Persistent chronic inflammation due to infections or poor homeostasis creates a favorable environment for cancer growth [34]. Cyclooxygenase-2 (COX2), an enzyme that promotes inflammation by inducing the synthesis of prostaglandins, has been found to be overexpressed in the tumor microenvironment [35]. Non-steroidal anti-inflammatory drugs (NSAIDs), a class of commonly used medications for the treatment of pain and inflammation, are well known as COX inhibitors. NSAIDs have been shown to impede both COX-dependent and -independent tumor-related inflammatory processes [36], and their combination with anticancer drugs has been explored to restrict tumorigenesis [37].

### 2.5. Reduced Toxicity

Combination therapy allows the use of lower drug quantities as a direct consequence of their synergetic action. This reduces the intensity of the toxicities associated with anticancer therapy while the probability of therapy success increases. A clinical trial on the combination of Chinese herbal medicine (PHY906) with capecitabine revealed a lowering of the treatment-related toxicities, with higher therapeutic outcomes [38].

### 2.6. Improved Tumoral Drug Distribution

The poor efficacy of anticancer drugs is often linked to their inadequate penetration into solid tumors. Tumor-specific barriers such as high interstitial fluid pressure, dense extracellular matrix (ECM), abnormal vasculature, and active drug efflux mechanisms hinder drug diffusion and retention within the tumor microenvironment [39]. Anticancer drugs have been shown to markedly reduce tumor growth when combined with drugs that promote their tumor penetration. Nitric oxide (NO) donors can dilate blood vessels, increasing perfusion and enhancing drug penetration. A combination of *tert*-dodecane S-nitrosothiol (NO donor) with doxorubicin (DOX) resulted in elevated levels of intracellular DOX by decreasing the endosomal membrane integrity, and hence, enhanced the anti-tumor effect of DOX against triple negative breast cancer [40]. The tumor ECM acts as a physical barrier that limits drug diffusion. Enzymes such as hyaluronidase (PEGPH20) break down hyaluronic acid, reducing the tumor stiffness and enhancing the penetration of small molecules and monoclonal antibodies. Administration of PEGPH20 with gemcitabine has shown improved efficacy in pancreatic cancer [41]. Tumor endothelial cells form tight junctions that prevent drug leakage into the tumor. Various agents that disrupt these junctions can facilitate the tumoral penetration of small-molecule drugs as well as macromolecular drugs [42,43]. Tumors actively expel chemotherapeutic agents via efflux transporters such as P-glycoprotein (P-gp). Inhibitors like verapamil and mifepristone block these efflux pumps, improving the intracellular retention of drugs like paclitaxel and doxorubicin [44,45].

## 3. Drug Delivery Systems for Multiple Drug Therapy

Anticancer treatment often faces several challenges in the absence of a rationally designed drug carrier. First and foremost is the indiscriminate distribution of the drug throughout the body. Even though most of the newly discovered molecules possess specificity for certain pathways, the administered drugs lack specific targeting, being passively distributed via the blood circulation. This affects the normal cells more than the cancerous ones, leading to severe life-threatening side effects that limit the overall therapeutic potential of the therapy due to the reduced tolerance for the administered dose [46]. Besides the uncontrolled biodistribution, the fate of the drugs after administration could be seriously affected in the absence of a suitable nanocarrier. The rapid metabolism and excretion of the drugs, coupled with uptake by the immune system, do not allow sufficient quantities of these molecules to reach the cancerous tissues [47]. Secondly, as the combination therapy is administered simultaneously or sequentially, different drugs reach the target tumor site at different rates owing to their physicochemical differences and pharmacokinetic fates [48]. As the principle of synergy is based on the optimal drug ratio at the target site to target multiple pathways or mechanisms, the lack of a proper drug carrier often results in inconsistent drug ratios in the cancer cells; thus, it manifests in contradictory and erratic outcomes [49]. Due to the rapid proliferation and mutations, cancer cells quickly adapt to the suboptimal survival stress imposed by combination anticancer therapy by developing alternate cellular pathways or reducing drug internalization due to rapid drug expulsion from the cells using efflux pumps [50]. Therefore, without a suitable drug carrier, there is a differential distribution of the anticancer drugs within the tumor mass, which increases the chances of survival of some cells, thereby increasing the likelihood of resistance.

Despite the fact that a well-designed drug combination may provide superior anticancer results in vitro, the above-mentioned factors render the therapy useless in a real-life complex disease state without the use of an advanced drug delivery system (DDS). The use of nanotechnology has proven to be an effective way to deal with these issues. A plethora of nanoparticles are under investigation for biomedical applications, such as micelles, lipid nanoparticles, dendrimers, polymer–drug conjugates, nanocages, and many more [51]. Among the most studied nanosystems used for carrying the drug to the target organ/cell, the formulations made from lipids are the most promising ones, having a high translation potential from bench to bedside due to their high resemblance to the cell membrane. Liposomes are particularly well suited for multidrug encapsulation due to their unique structural and functional properties [52]. Their aqueous core allows the encapsulation of hydrophilic drugs, while the lipid bilayer provides an ideal environment for hydrophobic drugs, enabling the simultaneous delivery of diverse therapeutic agents [53]. Additionally, their modifiable surface properties permit functionalization with targeting ligands, enhancing tumor selectivity and reducing systemic toxicity [54]. The high biocompatibility and biodegradability of liposomes make them a safer alternative to synthetic polymeric nanoparticles, and their ability to control drug release kinetics ensures optimized therapeutic efficacy [55].

This manuscript focuses on a critical niche of “microfluidics for liposomal co-encapsulation”, which is under-represented but highly relevant to advancing cancer treatment. By addressing the gap in knowledge, this manuscript brings significant value to the field. While breakthrough advances may be limited, this highlights the importance of this manuscript in addressing unmet needs and driving innovation.

### 3.1. Liposomes

A wide range of nanosystems have been utilized for the delivery of active pharmaceutical ingredients; however, liposomes are the oldest [56] and the most commonly used drug nanovehicles [57,58]. Liposomes are nanosized vesicular systems made up of a variety of phospholipids. They possess a high degree of biocompatibility, greater control over the drug release behavior, and the unparalleled ability to encapsulate both hydrophobic and hydrophilic drugs [59]. Liposomes can be easily modified or tailored to the needs of the therapy, thus reducing the side effects by minimizing the drug exposure in unrelated organs [60].

**Figure 1:**
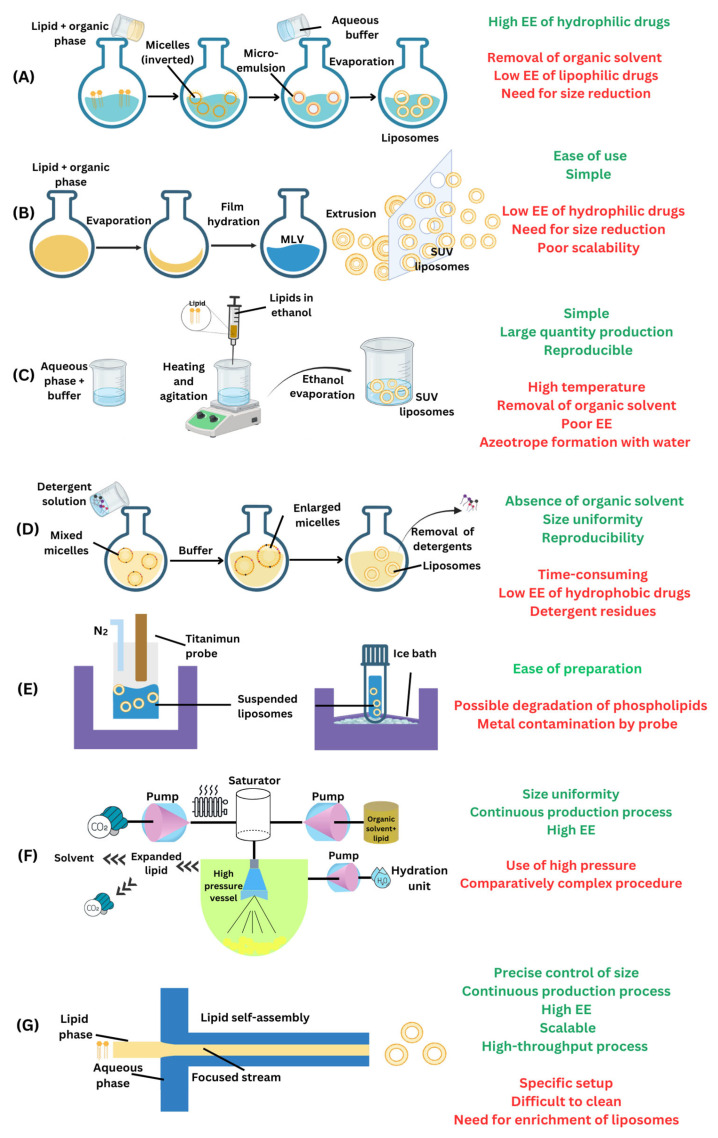
Scheme of the preparation of liposomes via various techniques along with their advantages and disadvantages: (**A**) reverse phase evaporation method; (**B**) thin-film hydration technique; (**C**) ethanol injection method; (**D**) detergent removal technique; (**E**) sonication process; (**F**) supercritical fluid technique; and (**G**) microfluidics approach. Abbreviations: EE, encapsulation efficiency; MLV, multilamellar vesicle; SUV, small unilamellar vesicle.

Liposomes are prepared using phospholipids as building molecules due to their ability to self-assemble into spherical nanosystems under certain experimental conditions [53]. Depending on the preparation method, the resulting nanovesicles are more or less homogeneous, which could imply the need for additional steps to improve the size distribution and unilamellarity [61]. For instance, the most commonly employed preparation method, thin-film hydration (TFH), is a very simple discontinuous approach, but extrusion is mandatory to obtain unilamellar liposomes with a narrow size distribution [62]. In addition, the low encapsulation efficiency together with the waste of phospholipids and the quite difficult removal of organic solvents used to dissolve the phospholipids are serious impediments to TFH [63]. Different preparation techniques for liposomes, with their advantages and disadvantages, are illustrated in Figure 1. To provide a clearer comparison between microfluidic and conventional techniques, we have also included Table 2. Overcoming the drawbacks of the THF approach is possible when liposomes are prepared in a confined space, as in the case of the microchannels of a microfluidic device. This continuous flow technology changes the paradigm of liposome manufacturing to ensure the optimum properties of the final liposomal formulations.

### 3.2. Exploring Microfluidics for Co-Encapsulation of Antineoplastic Agents

Microfluidics uses a high surface-to-volume ratio at the microscale to enhance the liquid manipulation, significantly improving the energy and mass transfer [64]. The rapid fluid mixing in microfluidics allows precise control over the key properties of liposomes: lamellarity, size, and uniformity [65]. In contrast, conventional methods like TFH or ethanol injection cause slow lipid displacement, leading to polydisperse liposome suspensions with varying compositions, structures, and sizes [66]. The reproducibility offered by the microfluidics minimizes the batch-to-batch variability [67]. Due to the controlled and confined mixing of the fluids in a microfluidic chip, both hydrophilic and hydrophobic drugs can be actively encapsulated with superior efficiency as compared to other methods, which require passive drug-loading approaches [68]. Microfluidics can be easily scaled up for industrial demands [69] and enables the production of a large number of doses of liposomes as a highly efficient throughput process [70]. Continuous-flow synthesis by microfluidics is an energy-efficient method for producing a high yield of liposomes with lower reagent consumption, hence minimizing the production costs [71]. In addition, microfluidics presents the possibility of real-time production monitoring and control of the parameters for maintaining the quality profile of the liposomes [72].

#### 3.2.1. Technical Considerations

The understanding of the microfluidic process variables (e.g., microfluidic channels’ geometry (shape, depth, width, aspect ratio, length, and layering), flow rates, temperature) and material attributes (nature and content of phospholipid and organic solvent, pH, ionic strength, and composition of the aqueous phase) is pivotal to obtaining liposomes with desirable characteristics. The liposome preparation process plays a crucial role in determining the drug-loading capacity, encapsulation sequence, drug ratio, and release kinetics [61].

##### Micromixer Geometry

The size and polydispersity of liposomes depend upon the efficiency of mixing the lipid phase with the aqueous phase, and the geometry of the microchannels in a micromixer that produces higher mixing efficiencies at a rapid rate is preferred. Microfluidic hydrodynamic chips with different angles of inlet have been reported to fabricate liposomes with variable sizes and distributions. A 45° inlet geometry produced a less hydrodynamically focused ethanol stream than a 90° inlet structure. In addition, the change in the total fluid flow rate affected the liposome size due to the perpendicular architecture, but not the liposomes produced by a 45° chip [73]. The aspect ratio of the microchannels in a hydrodynamic flow-focusing micromixer was found to be directly related to the size of the liposomes and inversely related to the size distribution. An approximately six-fold increase in the liposome production rate was also observed for the highest aspect ratio micromixer [74].

Ota et al. compared the physicochemical properties of the liposomes prepared via a static mixer and a staggered herringbone mixer. Significantly smaller liposomes with a broader size distribution were obtained by a staggered herringbone static mixer produced larger liposomes that were monodispersed in size, revealing the more controlled production capability of its geometry [75]. Bing and coworkers investigated the mixing efficiency of an S-type multi-cycle staggered herringbone mixer and noticed that at low a Reynolds number (Re), e.g., less than 10, the inlet shape has a negligible effect on the mixing; however, Y-inlets may be preferred to account for the pressure drop. Moreover, the 50 µm notch depth of the herringbone grooves was found to be suitable for rapid mixing with greater efficiency and a lower pressure drop. The herringbone structure induced chaotic mixing, resulting from the higher secondary flow intensity in the microchannels [76]. Recently, Ceccato et al. demonstrated that the 3D multihelical micromixer allows complete mixing at a high total flow rate (TFR) and efficient mixing at a low TFR. The secondary flow generated by the helical structure of the microchannels, even at lower flow rates, results in chaotic advection that permits sufficient mixing for the fabrication of liposomes [77].

##### Flow Rates

The TFR and flow rate ratio (FRR) are crucial in the mixing of the fluids that govern the time for precipitation and nucleation of the lipids within the microfluidic devices. Shah et al. revealed that a higher TFR and FRR allowed rapid fluid mixing, leading to quick precipitation of the phospholipids and production of smaller liposomes in a staggered herringbone mixer [78]. The TFR and FRR were also both observed to influence the liposomes’ size in a periodic mixer operating on the principle of Dean flow dynamics. The TFR had a more prominent impact than the FRR on the size, whereas the zeta potential (Z.P.) of the liposomes was independent of the flow rates. The polydispersity index (PDI) was only affected at low FRR values, e.g., below 3 [79]. The design of the micromixer can offset the influence of changes in the flow rates, as mentioned by Weaver et al., as the changes in the TFR were offset by the presence of internal structures within the microchannels [80].

##### Lipid Composition and Choice of Solvent

The self-assembly of phospholipids to generate liposomes is affected by the type, concentration of phospholipid, and percentage of cholesterol in the total lipid. For instance, an increase in the size and PDI of liposomes with an increasing concentration of phospholipid content was reported by Shah and colleagues. However, an increase in the cholesterol percentage resulted in greater increments in the size and PDI of the liposomes [78]. The ability of liposomes to encapsulate drugs is influenced by their lipid composition and the method of preparation. Hydrophilic drugs are sequestered in the aqueous core, whereas hydrophobic drugs integrate into the lipid bilayer [81]. Cholesterol improves the stability of liposomes and the encapsulation of hydrophobic drugs within the lipid bilayer, but a relatively higher cholesterol content also leads to reduced space for the hydrophobic drugs in the bilayer [82]. Lopez et al. revealed that the carbon chain length of the phospholipids could impact the size of the liposomes more than their concentration, as the lipid transition temperature depends on the number of carbons in the backbone of the lipid. Therefore, the temperature increase also decreases the size of the formed liposomes. Moreover, they iterated that the Z.P. depends on the terminal polar group of the phospholipid [79]. Choi et al. showed that in a flow-focusing geometry, the addition of a short acyl chain phospholipid could change the membrane properties of the liposomes by inducing heterogeneity and decreasing the size, whereas cholesterol increases the membrane rigidity and induces anisotropy, hence increasing the size of the liposome. They also observed that using ethanol as a solvent instead of isopropyl alcohol (IPA) produces smaller liposomes, owing to ethanol’s reduced viscosity and ability to rapidly diffuse into the aqueous phase as compared to IPA [83]. Recently, Maeki et al. studied the effect of the ethanol concentration on the liposomes and noticed temporal changes in the liposome structure. Ethanol induced multilamellarity in unilamellar liposomes and increased the bilayer d-spacing in a concentration-dependent manner. Hence, a microfluidic system integrated with the ethanol removal from the liposomal suspension will decrease the structural impact of ethanol on liposomes and improve the throughput of the technique [84].

##### Drug Encapsulation Method

The method used for drug loading in liposomes, especially hydrophilic drugs, dictates the amount of drug being encapsulated and the physicochemical properties of the drug-loaded liposomes. Encapsulation of water-insoluble drugs is favored within the hydrophobic bilayer assembly. However, the same bilayer is responsible for the lower loading capacities of liposomes in the case of hydrophilic drugs, as it obstructs the entry of the drug within the aqueous core of the liposomes from the exterior hydrophilic bulk [85]. Drug-loading techniques can be broadly characterized as passive loading and active loading methods. The passive loading technique can be termed an in-line loading method as the drugs are loaded during the preparation of liposomes, such as a hydrophilic drug dissolved in the aqueous phase and a hydrophobic drug dissolved in the lipid-containing organic solvent [86]. Passive loading works well for hydrophobic drugs since they stay in the bilayer [87]. For ionizable hydrophilic drugs, active or remote loading is preferred as it allows drug partitioning within the aqueous core of the preformed liposomes based on the pH or ion gradient. The neutral drug permeates the bilayer membrane and becomes ionized in the core of the liposomes, restricting its outward movement and ensuring reasonable encapsulation efficiency [88]. Pisani et al. compared different active loading methods (pH gradient, freeze–thaw method, sonication, electroporation) for the encapsulation of proteins in liposomes synthesized by means of the microfluidic technique. Electroporation markedly increased the BSA encapsulation but at the expense of a massive size increase (more than 600 nm). The freeze–thaw method was considered most suitable for its ability to improve the protein-loading capacity of liposomes while retaining the smaller vesicle structure below 200 nm [89]. The order in which the drugs are introduced during liposome formation affects their spatial localization within the vesicle. Sequential loading can lead to different partitioning patterns between the aqueous core and lipid bilayer. Nam et al. showed that sequential loading of DOX in erlotinib (ERL)-loaded liposomes was favored by the ammonium sulfate gradient method and not by the pH gradient technique [90]. Recently, Jaradat et al. also noticed that polyethylene glycol (PEG) functionalization of liposomes (PEGylation) decreases the size, hence limiting the expansion of the aqueous core and resulting in reduced accommodation of the passively loaded hydrophilic carboplatin [91].

Given the importance of achieving the efficient co-encapsulation of multiple drugs at optimal ratios, the key strategies are summarized in Box 1 to provide a practical framework for optimizing microfluidic systems to enhance the therapeutic outcomes in co-encapsulated liposome formulations.

Box 1 **Strategies for the efficient co-encapsulation of multiple drugs in liposomes by microfluidics.**

1. Optimization of microfluidic parameters
  Microfluidic techniques allow precise control over mixing, flow rates, and liposome formation, which is essential for co-encapsulation.
            i. Flow rate ratio (FRR):

- Adjusting the ratio of the lipid-containing organic phase to the aqueous phase influences the liposome size and drug encapsulation efficiency. Higher FRRs enhance lipid precipitation, promoting simultaneous encapsulation of hydrophilic and hydrophobic drugs into the aqueous core and lipid bilayer, respectively.
            ii. Total flow rate (TFR):
- Increasing the TFR improves mixing efficiency, leading to a uniform drug distribution within liposomes. TFR adjustments can help fine-tune drug loading while minimizing variability.
            iii. Microfluidic mixer geometry:
- Mixers with optimized channel geometries (e.g., staggered herringbone or chaotic mixers) enhance the uniform distribution of both drugs during liposome synthesis.
- Rapid mixing at microfluidic junctions ensures simultaneous encapsulation without competitive displacement.

2. Use of drug-specific partitioning
  The physicochemical properties of the drugs dictate their partitioning between the aqueous core and the lipid bilayer during co-encapsulation.
            i. Hydrophilic and hydrophobic drug combinations:

- Hydrophilic drugs are encapsulated within the aqueous core, while hydrophobic drugs partition into the lipid bilayer.
- Proper solubilization of both drugs in their respective phases before mixing is critical to achieving the desired ratio.
- Solvents with high miscibility (e.g., ethanol) are preferred for the lipid phase to achieve efficient hydrophobic drug encapsulation and maintain the integrity of the aqueous drug.
            ii. Lipid composition:
- Using lipids with optimal hydrophobicity and flexibility enhances bilayer stability and loading capacity for hydrophobic drugs.
- Incorporating cholesterol increases membrane rigidity, improving the retention of both drug types.

3. Remote loading for dual drugs

- This technique utilizes preformed liposomes with gradients (e.g., pH, ion, or charge) to actively load both drugs after liposome formation.
- A pH gradient can drive hydrophilic drug molecules into the aqueous core. Simultaneously, lipophilic molecules interact with the lipid bilayer.

##### Drug Release Behavior

Drug release is one of the crucial factors that governs the course and outcome of the therapy. It also reflects the stability of the formulation and defines the shelf life of the product. A formulation that retains the drug during storage but releases the drug in a controlled and predictable way in the body is highly desired by scientists [92]. In cancer management applications, the desirability of an optimum release profile is quite high. The premature leakage of drugs from nanostructured carriers during their circulation or within normal tissues results in a form of severe toxicity and sub-therapeutic levels in cancerous tissues, which negatively affect the decision to continue the therapy [93]. The situation becomes worse if the nanocarrier encapsulates multiple drugs. Simultaneous release only in the vicinity of cancerous tissues or within the tumor will serve the purposes of coadministration, such as synergistic therapeutic action, reduced side effects, minimal chances of resistance development, lower dosing frequency, and improvements in the patient’s quality of life [94]. Sequential release of the co-encapsulated antineoplastic agents could also be sought if it improves the therapy [95].

The release of encapsulated drugs from the liposomes is influenced by multiple factors that are intertwined with each other [96]. The physicochemical properties of loaded pharmaceuticals can provide an idea about their release from the liposomes in different physiological environments [97]. The type of phospholipids [98], amount/concentration of cholesterol [82], presence of charged lipids [99], and degree of PEGylation have been found to play their part in controlling the drug’s release from liposomes. The size, size uniformity, fluidity, and lamellarity of the liposomes have been reported to impact the drug release behavior [100].

Incorporating cholesterol increases membrane rigidity, reducing the premature leakage of hydrophobic drugs as it improves the interaction of these drugs with the long chains of phospholipids. However, it promotes the rapid release of hydrophilic molecules as cholesterol’s presence decreases their favorable hydrophilic interaction with polar phosphate headgroups of phospholipids [82]. The presence of a hydrophobic drug has also been found to negatively affect the diffusive barrier properties of liposomes, which could cause faster release of water-soluble drugs [101]. Passive drug release is predominant from liposomes owing to their inherent structural characteristics. Tailoring the drug release profile can be achieved through the inclusion of stimuli-responsiveness during the design of liposomes. These stimuli could be internal (pH change [102], enzymes in the tumor microenvironment [103], or reactive oxygen species (ROS) [104]) or external (temperature [105], light [106], or ultrasound [107]). Pilkington et al. demonstrated that microfluidics can be used to design multicompartmental liposomes encapsulating multiple cargoes, and the release of each encapsulated molecule can be modulated separately by integrating a unique responsiveness feature into each compartment [108]. Overall, incorporating responsiveness to various stimuli increases the liposomal therapeutic efficiency and would be a significant attribute in the design of MDT.

##### Long-Term Storage Stability

The long-term stability of liposomal formulations is critical to their clinical translation and commercial viability. Liposomes are prone to physical, chemical, and biological instability, which can lead to aggregation, leakage of the encapsulated drug, oxidation, and degradation of lipid components over time. Ensuring stability during storage is essential to maintaining therapeutic efficacy, safety, and regulatory compliance [109]. Over time, liposomes tend to aggregate, fuse, or undergo phase separation, altering their size distribution and affecting the drug release kinetics [110]. Changes in the temperature and ionic strength can further accelerate these processes, compromising the product integrity [111]. The phospholipids used in liposome formulations, particularly unsaturated lipids, are highly susceptible to oxidation when exposed to oxygen, light, or free radicals. The presence of peroxide impurities in lipids also makes them prone to oxidative stress [112]. The hydrolysis of ester bonds in phospholipids also generates free fatty acids and lysophospholipids [113]. Both the oxidation and hydrolysis of lipids lead to membrane degradation, causing increased permeability and drug leakage, and contribute to a reduced shelf life [98]. Additionally, macromolecular drugs may undergo chemical degradation within the liposome, leading to the formation of inactive or toxic byproducts [113].

For liposomal drugs to be approved for clinical use, they must meet strict regulatory stability requirements established by the Food and Drug Administration (FDA; Silver Spring, MD, USA) and European Medicines Agency (EMA; Amsterdam, Netherlands). Stability studies must demonstrate acceptable physicochemical properties, drug retention, and sterility over the intended shelf life [114]. One of the most effective methods for enhancing long-term stability is lyophilization (freeze-drying), where water is removed to convert liposomes into a stable dry powder. This method prevents aggregation, oxidation, and hydrolysis, extending the shelf life significantly. However, the freeze-drying process can lead to membrane damage, necessitating the use of cryoprotectants, such as sucrose, trehalose, and glucose, which prevent lipid fusion and maintain vesicle integrity [115]. The choice of lipids plays a crucial role in determining liposomal stability. Formulations containing saturated phospholipids (e.g., DSPC, DPPC) and cholesterol exhibit greater stability than those using unsaturated lipids, as they are less prone to oxidation and hydrolysis [53]. Additionally, PEGylation improves steric stabilization, preventing aggregation and fusion over time [91]. Different polymeric coatings have also been evaluated to improve liposomal stability and augment biological performance [116]. Liposomal formulations should ideally be stored at low temperatures (2–8 °C) and a neutral pH to minimize hydrolysis and oxidation. Refrigerated storage delays lipid degradation, while proper buffer selection ensures pH stability [109]. For some formulations, storage at −80 °C further enhances the stability [117]. To prevent oxidative degradation, antioxidants such as α-tocopherol (Vitamin E), butylated hydroxytoluene (BHT), and ascorbic acid are often incorporated into liposomal formulations [112]. These compounds scavenge free radicals, protecting the lipids from oxidation and extending the shelf life.

#### 3.2.2. Microfluidic-Assisted Design of Antineoplastic Agents Co-Encapsulated in Liposomes

**Figure 2:**
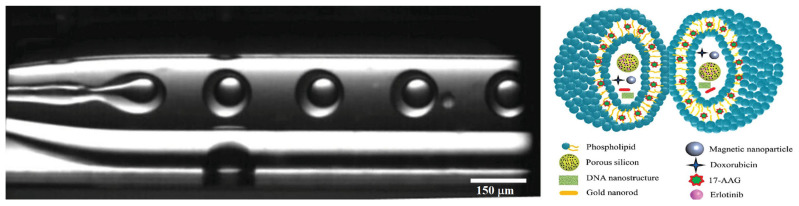
Optical microscopy image depicting the synthesis of photothermal-responsive giant magnetic liposomes encapsulating Psi NPs, drugs (doxorubicin, 17-N-allylamino-17-demethoxygeldanamycin (17-AAG), Erlotinib), gold nanorods, and DNA nanostructure via a double emulsion (w/o/w) microfluidic device (reprinted from [118], with the permission of John Wiley and Sons Limited).

Due to the complexity of the tumor and the rapid development of resistance, a cocktail of active molecules was encapsulated within giant micron-sized liposomes using a w/o/w double emulsion approach via a glass capillary microfluidic device, as shown in Figure 2 [118]. The hydrophobic 17-N-allylamino-17-demethoxygeldanamycin (17-AAG) was encapsulated within the phospholipid double layer, while the aqueous core contained the hydrophilic DOX, negatively charged photothermal-responsive porous silica nanoparticles loaded with hydrophobic Erlotinib (−35 mV and 171 nm), gold nanorods with an almost neutral surface charge (−0.6 mV and 50 nm), positively charged nano-magnetite (7 mV and 15 nm), and 25 nm stripes of DNA nanostructure (DAO-E AB). This micro-in-nano hybrid platform was evaluated for the synergistic efficacy of the combinations and its ability to inhibit drug resistance in the DOX-resistant breast cancer cell line MCF-7/DOX. DNA nanostructures were included to sensitize the drug-resistant cancer cells, whereas gold nanorods not only improved the loading of DOX (93% EE) and DNA nanostructures (94% EE) but also imparted thermo-responsiveness so that the drug release could be modulated at the target site. The encapsulation efficiency (EE) for 17-AAG was also observed to be greater than 90%. The absence of burst release for the encapsulated therapeutic agents was noticed. The DNA nanostructures and DOX release were more than 80% within 24 h, whereas only 50% Erlotinib was released at the same time. Hyperthermia from the gold nanorods increased the DOX release, but its effect on other drugs was not reported. The blank nano-in-micro platform was found to be cytocompatible with different cell lines (HeLa, M28, MCF-7, and MCF-7/DOX) up to a concentration of 500 µg/mL. For MCF-7/DOX cells, a higher number of dead cells was observed for the DNA nanostructures and multiple drugs co-encapsulated in giant liposomes than for the same drug combinations at the same ratios but without DNA nanostructures. The study showed promising in vitro results, but the rationale for the chosen drug combination and ratios was unclear. The impact of different liposome compositions and properties on biological performance was not explored, and the magnetic responsiveness of the nano-magnetite-loaded liposomes was not evaluated. In vivo studies on the thermo-responsiveness, magnetic responsiveness, hemocompatibility, safety, pharmacokinetics, biodistribution, and efficacy in tumor-bearing models are needed to assess their real-life performance.

Gkionis et al. studied how different parameters of a microfluidic method using a flow-focusing micromixer affect the preparation of PEGylated liposomes co-loaded with hydrophilic DOX and hydrophobic umbelliprenin, comparing it to the thin-film hydration technique [119]. The loading of both drugs increased the liposomes’ size from both methods. When using the active loading method by means of thin-film hydration, the EEs for DOX and umbelliprenin were >90% and 29%, respectively, whereas the microfluidic method produced liposomes with EEs of 74% and 47% for DOX and umbelliprenin, respectively. A stability study at 4 °C over a period of 4 weeks showed a minimal increase in the size and PDI of the formulations synthesized using both methods, indicating stable liposomes. Up to 48 h, only 30% DOX release was observed for thin-film hydrated liposomes as compared to the 90% DOX from the microfluidic liposomes. The authors did not report the release of umbelliprenin and its effect on the release of DOX. Blank liposomes did not show toxicity toward the three tested breast cancer cell lines (MCF-7, MDA-MB 231, and BT-474). The free DOX solution had a lower half-maximal inhibitory concentration (IC_50_) than the co-loaded liposomal formulations. Microfluidic liposomes exhibited higher cytotoxicity than thin-film liposomes, attributed to the differing DOX release rates. However, a comparison with a mixture of free DOX and umbelliprenin was not provided to validate the drug combination. While umbelliprenin’s roles in vesicle stabilization and MDR reversal were discussed, co-loaded liposomes were not tested in drug-resistant cancer cells to confirm their efficacy. Additionally, the impact of PEGylation on the liposomes’ physicochemical properties and anticancer activity was not evaluated.

**Figure 3:**
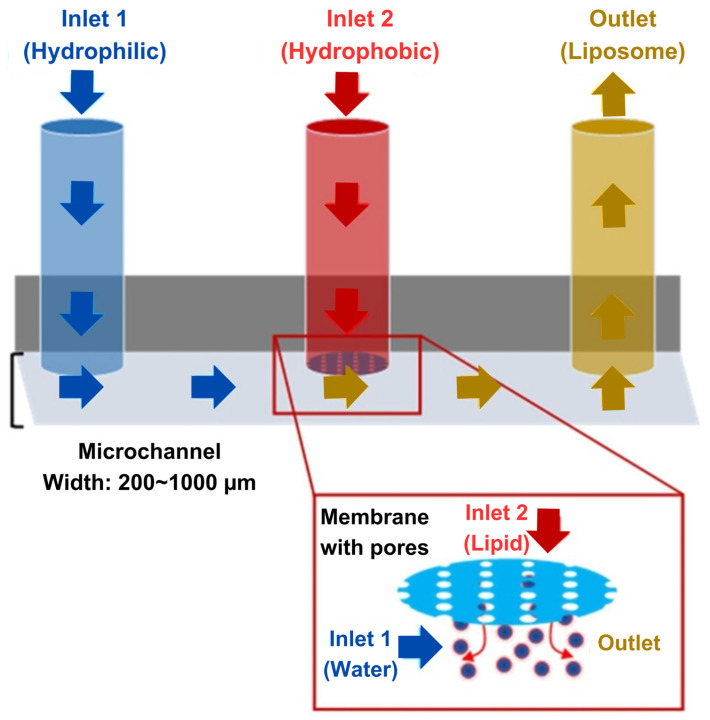
Schematics of the multi-hydrodynamic focusing device made of two vertically combined polydimethylsiloxane layers with a microporous stencil between the passage of the lipid inlet and tge microchannel for the fabrication of catechin- and curcumin-loaded liposomes (reprinted from [124], with permission from Elsevier).

Curcumin is a naturally occurring flavonoid with excellent anticancer potential against many cancer types [120,121,122]; however, its activity has not been clinically translated due to its hydrophobic nature and poor bioavailability owing to the rapid degradation and efflux mediated by p-glycoprotein [123]. A microfluidic hydrodynamic focusing device, as shown in Figure 3, comprising a couple of vertically combined layers of polydimethylsiloxanes, was used by Hong et al. for the fabrication of liposomes co-loaded with hydrophilic catechin and hydrophobic curcumin to improve the overall anticancer effect [124]. Monodisperse spherical liposomes were obtained with a negative surface charge (−30 mV), which was then reversed by the addition of a cationic surfactant (hexadecylamine) for better cell penetration. A 100% EE was achieved for curcumin but a mere 16% of the catechin was encapsulated in dual-loaded cationic liposomes. The cytotoxicity studies against colon cancer cells (HT-29 and Caco-2) revealed dose-dependent effects for single drugs or combinations; liposomal encapsulation of single drugs also improved their efficacy. Nonetheless, the anticancer effects of dual-loaded cationic liposomes were much better. The study lacked comparison of negatively charged liposomes and omitted a preliminary assessment of the drug synergy. The poor catechin encapsulation may have hindered achieving the optimal drug ratio for synergistic effects. Additionally, the absence of drug release and permeation data limits the understanding of the bioavailability. The presence of free catechin with liposomal curcumin significantly impacted the cancer cell viability. Further data on the cellular uptake, p-glycoprotein efflux, biocompatibility, metabolic effects, and in vivo bioavailability are needed to evaluate the clinical potential of the formulation.

Radiotherapy is an effective way to kill tumors but the damage to normal tissues is difficult to avoid. To improve the safety and efficacy of radiotherapy, radiosensitizers have been exploited to reduce the level of radiation needed to kill tumor cells and to limit the effect of the radiation to the cancerous tissue only [125]. Charest and coworkers proposed the use of both types of therapy (chemotherapy and radiotherapy) in tandem by designing liposomes for the concomitant delivery of a chemotherapeutic agent and a radiosensitizer for enhanced therapeutic outcomes and an improved safety profile [126]. Carboplatin, an anticancer drug, was encapsulated along with the gold nanoparticles (GNPs) in the liposomes (LipoGold) made via microfluidics. Smaller liposomes (134 nm) with better control over the polydispersity (0.199 PDI) were obtained using cationic lipids in a herringbone micromixer. Low-dose LipoGold (1.44 µg/mL carboplatin and 1.07 µg/mL GNPs) after irradiation with X-rays (2 Gy; 80 keV) markedly reduced the survival of colon cancer cells (HCT-116) in an in vitro clonogenic assay. The effect was improved as compared to that obtained when a high dose of carboplatin, oxaliplatin, GNPs or their combinations with or without X-ray irradiation was employed. In a colorectal cancer model, intra-tumoral LipoGold injections significantly delayed tumor growth, comparable to a 69 times higher GNP dose with irradiation. This indicates that co-delivering the radiosensitizer and chemotherapy drug in liposomes enhances their effects by ensuring both reach the same location, which is harder to achieve with separate administration. However, the study lacks data on LipoGold’s physicochemical properties, such as the Z.P., and the release behavior of GNPs and carboplatin under various conditions. It also omits cellular and biochemical mechanisms, as well as biocompatibility and toxicity assessments.

Lv et al. loaded gold nanorods (GNRs), magnetic nanoparticles (MNPs), and DOX in a folic-acid-targeted liposome responsive to magnetic hyperthermia (thermosensitive liposomes; TML) with the aid of microfluidics for site-specific treatment of bladder cancer [127]. The aqueous phase containing GNRs (an approximate aspect ratio of 5.6 and a dimension of 41 nm × 7 nm) and DOX was mixed with the MNP (10 nm)-enriched lipid phase in a micromixing chip to obtain FA-TMLs@MNPs-GNRs-DOX liposomes. The blank liposomes were 95 nm in diameter and highly monodispersed (PDI = 0.02). Upon the addition of MNPs, GNR, and DOX, their size (234 nm) and polydispersity (PDI = 0.11) increased. At an optimal feed rate of 2.0 mg/mL DOX, 0.05 mg/mL MNPs, and 300 µM GNRs, the EE and DL of DOX in the FA-TMLs@MNPs-GNRs-DOX liposomes were 28.6% and 22%, respectively. The release of DOX from the liposomes proved to be temperature-sensitive (16% and 90% after 30 min at 24 °C and 45 °C, respectively). Irradiation of the FA-TML did not affect their toxicity profile, whereas irradiation decreased the cell survival from 97.6% to 74.4% for FA-TMLs@MNPs-GNRs. The viability of the bladder cancer cells additionally decreased from 29.7% to 7.4% upon irradiation of the FA-TMLs@MNPs-GNRs-DOX liposomes. Folate targeting was confirmed by the reduced cytotoxicity of the irradiated liposomes in folate-negative A549 cells (IC50 = 4.2 µM) as compared to the folate-positive bladder cancer cells 5637 (IC50 = 2.8 µM). Colocalization studies revealed the time-dependent uptake of FA-TMLs@MNPs-GNRs-DOX liposomes with a higher intensity of DOX up to 4 h in the nucleus of folate-positive bladder cancer cells. This study shows promising results but has several gaps. The MNPs were mixed with the lipid phase, yet their proposed distribution in the aqueous compartment with GNRs and DOX was not confirmed. The liposomes’ Z.P., crucial for cell interaction, was not reported. Drug release studies were not conducted at 37 °C, and the release of MNPs and GNRs was unexplored, potentially affecting the system performance. In vivo studies on the biodistribution, pharmacokinetics, and anti-tumor efficacy are required to evaluate the clinical potential of these DOX magneto-liposomal formulations.

**Figure 4:**
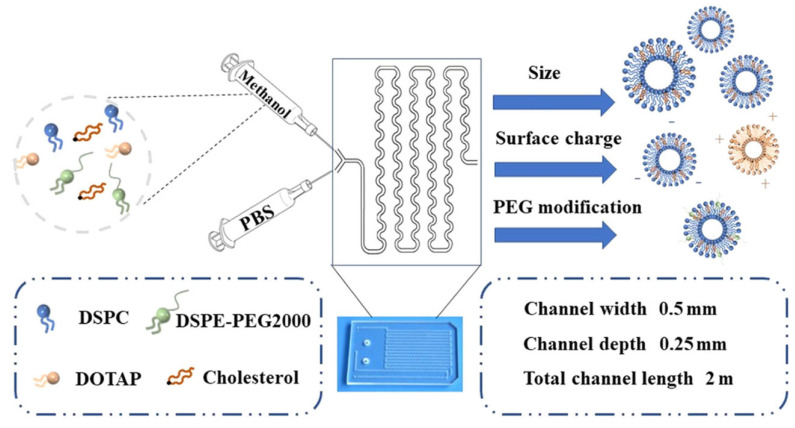
Schematics of the geometry of the micromixer used for the design of copper and chlorin e6 (Cu-Ce6) liposomes with diverse sizes, charges, and surface functionalization (reprinted from [128]. Copyright (2022) American Chemical Society). Abbreviations: DOTAP, 1,2-dioleoyl-3-trimethylammonium-propane; DSPE, 1,2-distearoyl-sn-glycero-3-phosphoethanolamine; DSPC, 1,2-distearoyl-sn-glycero-3-phosphocholine; PEG, polyethylene glycol; PBS, phosphate-buffered saline.

Li et al. loaded copper (Cu^2+^) for the modulation of the tumor microenvironment and chlorin e6 (Ce6) for photodynamic therapy within liposomes (Cu-Ce6@lip) by the microfluidic technique, as shown in Figure 4 [128]. Key liposomal properties were found to be significantly influenced by the changes in the FRR, TFR, lipid content, and degree of PEGylation. PEGylation reduced the surface charge and liposome uptake by HeLa cells, suggesting it helps prolong their circulation in the body without increasing their size. Reversal of the surface charge from negative to positive with the aid of 1,2-Dioleoyl-3-trimethylammonium propane (DOTAP) facilitated the uptake by HeLa cells, confirming the increased interaction of positively charged liposomes with the negatively charged cell membranes. Cu^2+^ was complexed with Ce6 through chelation to yield Cu-Ce6, which was confirmed by IR and UV spectroscopies. Cu-Ce6@lip (100 nm in size) scavenged the glutathione (GSH) and increased the ·OH levels due to the reduction of Cu^2+^ into Cu^+^ within the tumor environment. In addition, laser treatment increased the production of singlet oxygen species in a GSH-responsive manner. A higher uptake of Cu-Ce6@lip as compared to free Cu-Ce6 was noted in the HeLa cells. Biodistribution studies revealed higher localization of the Cu-Ce6@lip within the tumors than the Cu-Ce6, which was predominant in the liver. A significant reduction in the tumor volume was noticeable for the animal groups treated with Cu-Ce6@lip+laser compared to the animals treated with Ce6@lip+laser to confirm the synergistic role of Cu^2+^ and Ce6 in boosting photodynamic therapy. Phototherapy with Cu-Ce6@lip showed promising results; however, the EE and release of the Cu-Ce6 were not disclosed. The clinical relevance of Cu-Ce6 loading and the impact on the physicochemical properties of the final liposomal formulation was also not mentioned. The comparison of the Cu-Ce6@lip with the simultaneous administration of Cu@lip and Ce6@lip would have indicated the advantage of the co-loading approach.

**Figure 5:**
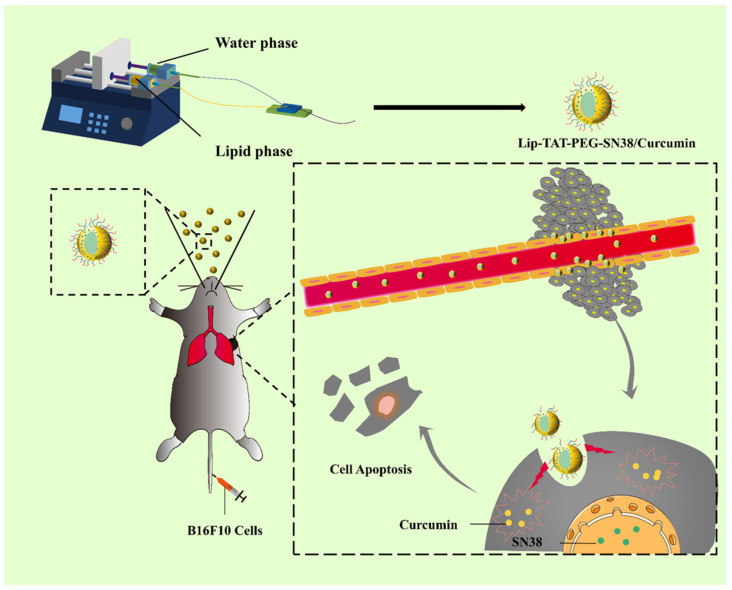
Schematic representation of the preparation of an amphiphilic camptothecin prodrug (TAT-PEG-SN38) linked to polyethylene glycol (PEG) and cell-penetrating peptide (TAT) co-encapsulated with curcumin in a liposome (Lip-TAT-PEG-SN38/Curcumin) and its effect in a mice model after pulmonary administration (reprinted from [131], with permission from Elsevier).

Pulmonary delivery of anticancer drugs for lung cancers via the inhalation or intranasal route seems more plausible as first-pass metabolism and untargeted drug distribution of the cytotoxic drugs can be avoided to improve the therapeutic outcomes and patients’ quality of life [129,130]. An amphiphilic camptothecin prodrug (TAT-PEG-SN38) linked to PEG and cell-penetrating peptide (TAT) was co-encapsulated with curcumin (CUR) in a liposome (Lip-TAT-PEG-SN38/CUR) for pulmonary delivery by Gao and colleagues, as presented in Figure 5 [131]. A Y-type micromixer was used to fabricate the dual-drug-loaded liposomes. The co-encapsulated liposomes (171 nm, PDI 0.124) were larger and more polydisperse than Lip-CUR (159 nm, PDI 0.110) but smaller than Lip-TAT-PEG-SN38 (222 nm, PDI 0.186). A higher EE for CUR was achieved by the microfluidics (88%) than via the ethanol injection technique (70%). Image J analysis showed that A549 cells took up Lip-TAT-PEG-SN38/CUR seven times more than Lip-CUR, likely due to TAT-enhanced permeation. The co-encapsulated liposomes induced significantly more A549 cell death than the unencapsulated CUR+SN38 combination. The CUR+SN38 and Lip-TAT-PEG-SN38/CUR displayed similar cell cycle arrest, but the apoptosis was 1.25 times higher for the co-encapsulated liposomes than for the free drug combination. Biodistribution studies post pulmonary administration revealed the initial distribution of the formulation in all the major organs, but after 24 h, the formulation was only noticeable in the lungs and liver. In vivo evaluation of the co-encapsulated liposomes in tumor-bearing mice revealed lower cell proliferation and reduced tumor nodes compared to other groups. Despite the promising results, there are some gaps in the study. The drug ratio was not based on preliminary biological studies, and the release and stability were not assessed in bronchial fluids. Furthermore, the benefits of co-encapsulation were not compared to separate liposomal preparations administered concomitantly. Jin et al. reported co-encapsulation of indocyanine green (ICG) as a biocompatible photosensitizer with the cytotoxic drug Ansamitocin P-3 (AP-3) to design a temperature-sensitive liposome (AI-TSL) utilizing the microfluidic swirl mixer [132]. The manufacturing parameters, such as the lipid concentration, TFR, FRR, and PEGylation degree, were optimized for the blank liposomes. The DL and EE for ICG were 1.6% and 51%, respectively, whereas the DL and EE for AP-3 were 4.4% and 80%, respectively. The size, PDI, and Z.P. of the spherical AI-TSL were 125 nm, 0.043, and −8.28 mV, respectively. The release of AP-3 was significantly higher from AI-TSL (89% within 12 h) upon laser irradiation and was attributed to the photothermal effect of ICG. The cytotoxicity results against breast cancer cells (MCF-7) further confirmed the results of the release experiment as the IC50 of AI-TSL in the absence of laser irradiation was 96.7 µM compared to the 16.8 µM upon laser treatment. In addition, the mixture of ICG and AP-3 with or without laser irradiation did not show much difference in the IC50 than the plain AP-3 solution, hence confirming the safety of the ICG. The tubulin depolymerization assay and cell signaling pathway assays confirmed the AP-3-mediated cell death, but the synergistic behavior of the AP-3 and ICG encapsulated liposomes (AI-TSL) at the cellular level was not elucidated. The EPR-mediated tumor distribution of AI-TSL was observed in the in vivo studies. Moreover, 97% suppression of tumor growth was observed for the laser-irradiated AI-TSL group, whereas a minimal toxic effect was observed by the blood cell counts as compared to the highly toxic free AP-3 solution. AI-TSL represents a promising and relatively safe delivery system for anticancer therapy; however, it lacks tumor targeting. The pharmacokinetic behavior was not studied, and the safety profiles need to be established for long-term administration in clinical settings.

**Figure 6:**
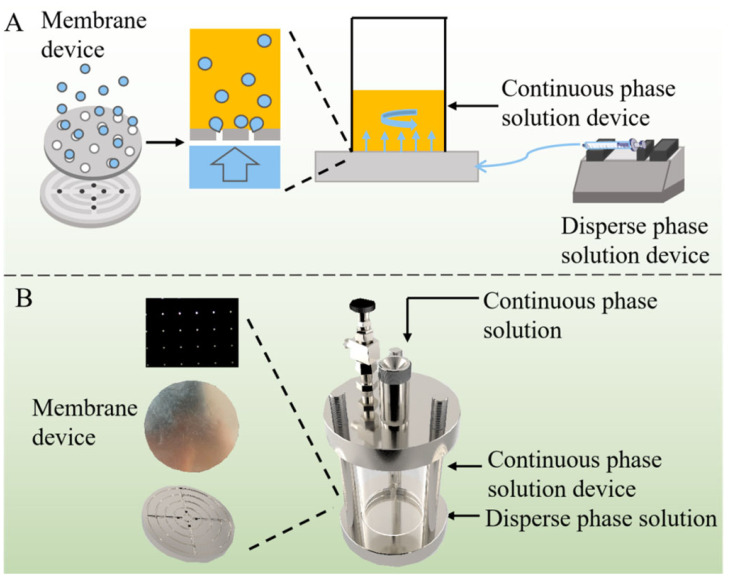
Schematic image of the microfluidic membrane emulsification platform for the manufacturing of nicotinamide mononucleotide and honokiol co-loaded liposomes. (**A**) Graphical presentation and (**B**) real-life image of a microfluidic device with a membrane and liquid distributor (reprinted from [133], with permission from Elsevier).

Recently, Zhang et al. developed a unique membrane emulsification coupled with microfluidic device (Figure 6) for the fabrication of liposomes co-loaded with a hydrophilic (nicotinamide mononucleotide (NMN)) and a hydrophobic drug (honokiol (HNK)) [133]. It was noted that the size and PDI of the liposomes could be manipulated by the injection rate of the phospholipid-containing organic solvent, the speed of mixing controlled by stirring, the distance between the stirrer and the membrane, the material used for membrane fabrication, the ratio of aqueous to organic phase, and the phospholipid concentration. Co-loading of NMN and HNK increased the size of the NMN@HNK@LNPs to 164 nm as compared to 110 nm for blank liposomes. The EE for NMN and HNK were 28.4% and 99.2%, respectively. NMN released rapidly (up to 3 h) from the liposomes and was found to be independent of the pH, whereas the HNK release was very slow (up to 96 h) and was relatively higher at the slightly acidic pH (5.5) than the neutral one. NMN@HNK@LNPs was found to be hemocompatible, but the cytotoxicity toward the cell was far greater than that of the HNK alone against HCT-16 cells, which was further confirmed by the higher cellular uptake of the co-loaded liposomes. The proposed liposome fabrication method is suitable for upscaling and continuous production, but the roles of the phospholipid type and organic solvent in emulsification require consideration. The effect of drug loading on the PDI and Z.P. was not addressed. The encapsulation efficiencies were given as percentages, while the release data were in mass, hindering performance correlation. Rapid NMN release compared to delayed HNK release may yield different clinical outcomes, with a higher likelihood of NMN loss in the systemic circulation before tumor targeting. Comparing co-loaded liposomes with single-drug-loaded ones in cellular studies could better demonstrate the co-loading effects and formulation efficiency.

Table 3 summarizes the liposomal formulations of the anticancer drugs obtained by microfluidic techniques alongside the key physicochemical properties of the co-loaded liposomes and the type of cancer for which they have been designed.

#### 3.2.3. Biological, Clinical, Safety, and Translational Considerations of Liposomal Cancer Therapy

While technical developments in liposomal drug formulations have improved their stability, loading capacity, and scalability, their ultimate success depends on their biological efficacy, immunogenicity, and clinical translation. Without a thorough understanding of how liposomes interact with the human body, even the most well-engineered formulations may fail in clinical applications. While liposomes have demonstrated immense promise in cancer therapy, addressing the challenges related to scalability, tumor heterogeneity, regulatory barriers, and personalized drug delivery is essential for their successful clinical translation. With advancements in microfluidic nanomedicine, targeted drug delivery, and combination immunotherapies, liposomes have the potential to redefine oncology treatment and improve patient outcomes in the near future. This section explores the critical aspects of advancing liposomal cancer therapies from laboratory research to real-world treatment.

##### Biological Efficacy Considerations

Liposomal carriers significantly improve tumor selectivity and drug accumulation due to the enhanced permeability and retention (EPR) effect, where the leaky tumor vasculature allows nanocarriers to accumulate in cancerous tissues while being retained due to poor lymphatic drainage [134]. However, the extent of the EPR-based accumulation varies across different tumor types, depending on factors such as the vascular density, interstitial pressure, and inflammation levels. While the EPR effect facilitates passive accumulation, many tumors exhibit heterogeneity in vascular permeability, which can lead to inconsistent drug delivery.

To overcome these limitations, researchers have developed multifunctional liposomes, integrating targeting, imaging, and immunomodulatory functionalities to optimize cancer treatment [63]. Coupling the controlled release properties of liposomes with tumor-homing will help maintain optimal drug ratios at the tumor site, preventing the sequential or inconsistent drug uptake often observed in conventional combination chemotherapy. For example, folate-modified liposomes selectively target folate receptors, which are overexpressed in ovarian and breast cancers [135], while HER2-targeted liposomes, conjugated with trastuzumab, enhancing drug uptake in HER2-positive breast cancer [136]. Similarly, cell-membrane-coated biomimetic liposomes can actively target glioblastomas to achieve improved brain tumor penetration [137]. Another emerging trend is the development of theranostic liposomes, which combine therapeutic and diagnostic functions in a single nanocarrier, enabling real-time monitoring of the drug distribution and treatment response. A multifunctional liposomal nanoplatform was developed by Shan et al. using a microfluidic mixing device (MMD) for PD-L1-targeted photoacoustic and fluorescence imaging, as well as photothermal therapy [138]. Such hybrid systems enable clinicians to monitor the drug biodistribution in real time, improving the treatment precision.

##### Immunogenicity and Safety Considerations

Although liposomes are widely regarded as biocompatible and biodegradable, their interaction with the immune system can sometimes trigger hypersensitivity reactions, accelerated clearance, or immune activation, limiting their therapeutic efficacy. One of the most well-documented immune reactions associated with liposomal therapy is complement activation-related pseudoallergy (CARPA), where liposomal formulations activate complement proteins, leading to pseudoallergic reactions such as hypotension, dyspnea, and anaphylactoid responses [139]. Moreover, upon intravenous administration, liposomes are subject to sequestration by the liver and spleen, where they are taken up by phagocytic cells of the mononuclear phagocyte system (MPS). While this uptake can be advantageous for treating hepatic malignancies, it can also lead to off-target accumulation and reduced drug availability at the tumor site [140]. The physicochemical properties of liposomes, including the size, surface charge, and lipid composition, play a crucial role in determining their pharmacokinetics, as larger liposomes tend to accumulate in the liver and spleen [141]. The use of PEGylation on the liposomal surface has been instrumental in extending the systemic circulation time of liposomes by shielding them from rapid clearance by the MPS. This stealth property enhances tumor accumulation and improves therapeutic outcomes [142]. However, repeated administration of PEGylated liposomes can lead to the production of anti-PEG antibodies, triggering the accelerated blood clearance (ABC) effect, which reduces the drug efficacy over time [143]. To address this issue, researchers are exploring alternative surface modifications, such as zwitterionic polymers and alternative coatings, which maintain prolonged circulation while minimizing immune recognition [144,145].

##### Clinical Considerations

The development of co-loaded liposomes using microfluidic techniques represents a significant advancement in cancer nanomedicine, offering precise control over the particle size, drug loading, and encapsulation efficiency (EE%), which are crucial for clinical applications. Nonetheless, it is crucial to design well-structured and rigorous clinical trials that include biomarker-driven patient stratification to validate the expected clinical outcomes, which include an enhanced therapeutic index, reduced MDR, improved tumor accumulation and drug penetration, minimized systemic toxicity, extended progression-free survival (PFS) and overall survival (OS), and improved patient compliance and quality of life.

To facilitate the clinical translation of co-loaded liposomes, a multi-phase trial approach is essential. Phase I trials should focus on evaluating the safety, pharmacokinetics (PK), and pharmacodynamics (PD) of the liposomal formulation, particularly assessing the drug release kinetics, systemic circulation half-life, and potential dose-limiting toxicities. Dose-escalation studies following a modified 3 + 3 design or Bayesian adaptive models could be employed to identify the maximum tolerated dose (MTD) while monitoring the immune responses and potential adverse events [146]. In Phase II trials, a biomarker-driven approach should be incorporated to evaluate the therapeutic efficacy in target patient populations, using genomic and proteomic markers to predict the response rates, such as the objective response rate (ORR) and progression-free survival (PFS) [147].

For Phase III trials, large-scale multi-center studies should be conducted to validate the clinical benefits, with a focus on the long-term survival outcomes. Head-to-head comparative trials would be crucial to demonstrate superior efficacy, reduced toxicity profiles, and improved patient compliance [148]. Additionally, real-world evidence (RWE) studies should be integrated to assess the long-term safety, patient adherence, and quality-of-life improvements [149]. Post-market surveillance in Phase IV trials should focus on identifying rare adverse events and evaluating the cost-effectiveness of microfluidic-based liposomal therapies in real-world settings. Pharmacovigilance programs should be implemented to monitor potential immunogenic responses, hypersensitivity reactions, and unexpected toxicities [150].

##### Translational Considerations

In 2017, a liposomal preparation of cytarabine and daunorubicin in a synergistic 5:1 molar ratio (VYXEOS) was approved by the FDA for treatment of acute myeloid leukemia (AML) or secondary AML with a poor prognosis. To date, it is the only combination nanomedicine available on the market. The treatment consists of an intravenous administration for 90 min on days 1, 3, and 5. The Combiplex platform was used for the selection of the synergistic molar ratios of the drug combination, aiming to reach the maximum efficacy and an efficient product development process [151]. Copper gluconate is utilized for the complexation of both drugs within the liposomes to ensure their retention in the liposomes, to avoid their premature leakage in the bloodstream, and to facilitate their simultaneous release in the bone marrow. This unique ensemble of old drugs has been reported to be more efficacious than the time-tested 3 + 7 strategy [152].

Microfluidics has been successfully used at an industrial scale to produce nanomedicines, and liposomes encapsulating nucleic acid prepared by microfluidics have already been approved by the FDA [153]. Pfizer-BioNTech utilized microfluidics to produce millions of doses of COVID-19 vaccine by using an impingement jet mixer [154]. The modular design of the microfluidic system with the parallel processing capability of multiple channels enables continuous production of liposomes. Hence, the loading of multiple antineoplastic agents in liposomes of uniform and desirable characteristics can be easily scaled up by employing microfluidics.

## 4. Challenges and Perspectives

Although multiple anticancer drug therapy has been beneficial in improving patient outcomes in most cases, the dream of winning the war against cancer is far from coming true. The benefits of combination therapy have not been fully realized due to the lack of an effective delivery vehicle to ensure the effective therapeutic actions of the drugs. The success of MDT not only relies on the identification of the synergistic dose combination of the anticancer modalities. It warrants the loading of multiple therapeutic entities within liposomes in the optimal ratios, the protection of the drugs by the nanocarrier within the systemic circulation, and the synchronous release at the target site in the same pattern as confirmed in preliminary efficacy studies. Furthermore, it is also worth noting that the main benefit of drug combination relies on the simultaneous release of the antineoplastic agents at the target site. Simultaneous passive loading of multiple drugs in the microfluidic mixers could not attain the required loading ratio; however, micromixers can be designed to integrate the remote loading of the drugs. The desired release behavior of the encapsulated drugs can be achieved by incorporating multifunctionality in the liposomes, such as stimuli-responsiveness to various internal (pH, reactive oxygen species (ROS), glucose levels, or enzymes) or external stimuli (temperature, sonication, or light).

Microfluidics enables high-throughput liposome production, but purification and concentration require dialysis or filtration to remove solvents and unloaded drugs. The correlation of manufacturing process parameters has been carried out with the variation of one parameter at a time in most studies. For industrial applications, the interplay of various parameters is more important for the desired attributes of the final product, and it provides a rich understanding of the underlying phenomenon. Optimizing micromixers for downstream integration could enhance microfluidics’ efficiency at an industrial scale. Moreover, in-line monitoring of the physicochemical aspects of the liposomes and loading efficiency determination would allow manipulation of the synthesis parameters during the production process without the need to stop the manufacturing operations. The cleaning of the microchannels also poses a significant challenge in the maintenance of the microfluidic devices. It is of prime importance to identify and avoid the fouling or clogging of the microchannels during the liposomal fabrication process to ensure efficient and uniform mixing throughout the synthesis scheme. In addition, the development of protocols to clean the microchannels would result in higher production cycles of microfluidic devices without the need to replace the micromixers.

Even though microfluidic-assisted liposome production holds great promise, there are many gaps that need to be bridged for the transition of bench-scale research to clinical application. A meager number of liposomal combination products for anticancer application have progressed beyond the research stage. There is a dire need for clear and concise regulatory guidelines for such complex systems, most importantly for manufacturing processes’ standardization and product quality control. The scaling up of liposome preparation via microfluidics for clinical applications has been perfectly demonstrated by the global supply of the COVID-19 mRNA vaccine produced by Pfizer-BioNTech. Nonetheless, there are still significant challenges associated with producing complex liposomes containing multiple drugs in a reproducible and scalable manner, which can be addressed through continued innovation in microfluidics.

## 5. Conclusions

As an advanced nanocarrier for multidrug therapy, liposomes provide an effective platform for the co-encapsulation of chemotherapeutic agents, ensuring synergistic action, enhanced bioavailability, and reduced systemic toxicity. Their adaptability for functionalization and controlled drug release profiles further solidify their superiority over conventional drug carriers in cancer treatment. In conclusion, co-encapsulated liposomes offer a significant paradigm shift from monotherapy to multiple drug therapy for the treatment of complex diseases like cancer. The microfluidics-based approach can transform cancer treatment by ensuring high therapeutic efficacy, lower side effects, and mitigation of drug resistance. Serious research efforts toward identifying the workable drug combinations, liposomal composition and functionalization, and microfluidics for the seamless production of LBDDS at an industrial scale are required to realize the full potential of anticancer MDT.

## Figures and Tables

**Table 1:** Combinations of drugs approved for the treatment of various cancers by the National Cancer Institute at the National Institutes of Health (Bethesda, MD, USA) [23].

Sr No.	Cancer	Drug Combinations
1	Acute Lymphoblastic Leukemia	Cyclophosphamide–Vincristine–Doxorubicin–Dexamethasone
2	Acute Myeloid Leukemia	Cytarabine–Daunorubicin–Etoposide
3	Bladder	Gemcitabine–Cisplatin
4		Methotrexate–Vinblastine–Doxorubicin–Cisplatin
5	Brain	Procarbazine–Lomustine–Vincristine
6	Breast	Doxorubicin–Cyclophosphamide
7		Doxorubicin–Cyclophosphamide–Paclitaxel
8		Doxorubicin–Cyclophosphamide–Fluorouracil
9		Methotrexate–Cyclophosphamide–Fluorouracil
10		Epirubicin–Cyclophosphamide–Fluorouracil
11		Doxorubicin–Cyclophosphamide–Docetaxel
12	Cervical	Gemcitabine–Cisplatin
13		Carboplatin–Paclitaxel
14	Chronic Lymphocytic Leukemia	Chlorambucil–Prednisone
15		Cyclophosphamide–Vincristine–Prednisone
16	Colorectal	Capecitabine–Oxaliplatin
17		Capecitabine–Irinotecan
18		Leucovorin–Fluorouracil–Irinotecan
19		Leucovorin–Fluorouracil–Irinotecan–Bevacizumab
20		Leucovorin–Fluorouracil–Irinotecan–Cetuximab
21		Leucovorin–Fluorouracil–Oxaliplatin
22	Endometrial	Carboplatin–Paclitaxel
23	Esophageal	Capecitabine–Irinotecan
24	Gastric	Leucovorin–Fluorouracil
25		Capecitabine–Irinotecan
26		Docetaxel–Cisplatin–Fluorouracil
27	Head and Neck	Carboplatin–Paclitaxel
28		Docetaxel–Cisplatin–Fluorouracil
29	Hodgkin Lymphoma	Ifosfamide–Carboplatin–Etoposide
30		Doxorubicin–Bleomycin–Vinblastine–Etoposide
31		Doxorubicin–Bleomycin–Vinblastine–Dacarbazine
32		Doxorubicin–Vincristine–Procarbazine–Prednisone
33		Mechlorethamine–Vincristine–Prednisone–Dacarbazine
34		Vincristine–Etoposide–Prednisone–Doxorubicin
35		Cyclophosphamide–Vincristine–Procarbazine–Prednisone
36		Cyclophosphamide–Vincristine–Procarbazine–Prednisone–Doxorubicin–Bleomycin–Vinblastine
37		Doxorubicin–Bleomycin–Vinblastine–Etoposide–Prednisone–Cyclophosphamide
38		Bleomycin–Etoposide–Doxorubicin–Cyclophosphamide–Vincristine–Procarbazine–Prednisone
39		Mechlorethamine–Doxorubicin–Vinblastine–Vincristine–Bleomycin–Etoposide–Prednisone
40		Vincristine–Doxorubicin–Methotrexate–Prednisone
41	Malignant Mesothelioma	Gemcitabine–Cisplatin
42	Multiple Myeloma	Bortezomib–Doxorubicin–Dexamethasone
43	Myeloproliferative Neoplasms	Cytarabine–Daunorubicin–Etoposide
44	Neuroblastoma	Busulfan–Melphalan
45		Carboplatin–Etoposide–Melphalan
46	Non-Hodgkin Lymphoma	Ifosfamide–Carboplatin–Etoposide
47		Rituximab–Ifosfamide–Carboplatin–Etoposide
48		Cyclophosphamide–Vincristine–Prednisone
49		Rituximab–Cyclophosphamide–Vincristine–Prednisone
50		Cyclophosphamide–Vincristine–Procarbazine–Prednisone
51		Cyclophosphamide–Doxorubicin–Vincristine–Prednisone
52		Cyclophosphamide–Vincristine–Doxorubicin–Dexamethasone
53		Etoposide–Prednisone–Vincristine–Cyclophosphamide–Doxorubicin
54		Rituximab–Etoposide–Prednisone–Vincristine–Cyclophosphamide–Doxorubicin
55		Rituximab–Cyclophosphamide–Doxorubicin–Vincristine–Prednisone
56	Non-Small-Cell Lung Cancer	Carboplatin–Paclitaxel
57		Gemcitabine–Cisplatin
58	Ovarian, Fallopian Tube, or Primary Peritoneal	Carboplatin–Paclitaxel
59		Gemcitabine–Cisplatin
60		Bleomycin–Etoposide–Carboplatin
61		Bleomycin–Etoposide–Cisplatin
62		Vincristine–Dactinomycin–Cyclophosphamide
63		Vinblastine–Ifosfamide–Cisplatin
64	Pancreatic	Gemcitabine–Cisplatin
65		Gemcitabine–Oxaliplatin
66		Leucovorin–Fluorouracil–Oxaliplatin
67		Leucovorin–Fluorouracil–Irinotecan–Oxaliplatin
68	Retinoblastoma	Carboplatin–Etoposide–Vincristine
69	Soft Tissue Sarcoma	Vincristine–Dactinomycin–Cyclophosphamide
70	Testicular	Bleomycin–Etoposide–Cisplatin
71		Vinblastine–Ifosfamide–Cisplatin
72		Etoposide–Ifosfamide–Cisplatin
73		Bleomycin–Etoposide–Carboplatin

**Table 2:** Comparison of microfluidic and conventional techniques for the synthesis of liposomes.

Feature	Microfluidic Techniques	Conventional Techniques (Thin-Film Hydration, Solvent Injection, Extrusion, Sonication)
**Size Control**	Excellent control (10–200 nm) with narrow size distribution due to precise fluid dynamics.	Size variability (50–500 nm) that requires post-processing (e.g., extrusion) for uniformity.
**Encapsulation Efficiency (EE%)**	Active loading is needed for the encapsulation of hydrophilic drugs.	Active loading is needed for the encapsulation of hydrophilic drugs.
**Reproducibility**	Highly reproducible due to automated, continuous-flow processing.	Batch-to-batch variability due to dependence on manual preparation and process conditions.
**Scalability**	Scalable via parallelized microfluidic chips and continuous-flow systems.	Limited scalability as batch-based production increases variability and processing time.
**Processing Time**	Rapid (< minutes per batch) continuous processing is possible.	Time-consuming (hours per batch) due to multiple steps (hydration, extrusion, filtration).
**Solvent Use and Removal**	Efficient solvent use with rapid removal; minimal residual organic solvents.	Require solvent evaporation, increasing processing time and potential toxicity risks.
**Clinical Feasibility**	Emerging in good manufacturing practice (GMP) manufacturing, potential for automated and sterile production.	Widely used in commercial formulations, but limited by batch-to-batch inconsistency.
**Cost & Infrastructure**	Requires specialized microfluidic chips and automation, the initial cost is high but decreases with scaling.	Lower initial costs, but higher long-term costs due to labor-intensive processing.

**Table 3:** The method of preparation, the composition of the liposomes, and the multiple antineoplastic agents co-encapsulated with liposomes prepared via the microfluidics approach against different cancers and their key physicochemical properties.

Sr. No.	Lipids	Hydrophilic Drug	Lipophilic Drug	Method	Size (nm)	Z.P. (mV)	PDI	EE (%)	Cancer	Ref
1	DPPC, DSPC, POPC, DOPC	Doxorubicin, Nanomagnetite, Gold nanorods, DNA nanostructures	17-AAG, Erlotinib	w/o/w double emulsion via glass capillary microfluidic device	30,000–50,000	–	–	–	HeLa, MCF-7, MCF-7/ADR, M28	[118]
2	DSPC, Chol, DSPE-PEG2000	Doxorubicin	Umbelliprenin	Hydrodynamic flow-focusing	227	−2.5	0.2	74 (Doxorubicin)/47 (Umbelliprenin)	MCF-7, MDA-MB 231, BT-474	[119]
3	DPPC, Chol, HDA	Catechin	Curcumin	Hydrodynamic flow-focusing	~200	~35	~0.2	16.2 (Catechin)/100 (Curcumin)	HT-29, Caco-2	[124]
4	DOTAP, DSPC, Chol	Cisplatin	Gold nanoparticles	Staggered herringbone micromixer	134	–	0.199	–	HCT-116	[126]
5	DPPC, Chol, DSPE-PEG2000, DSPE-PEG2000-FA	Doxorubicin, Gold nanorods	Magnetite nanoparticles	Microfluidic hybrid chip	234	–	–	28.6 (Doxorubicin)	Human bladder cancer cell line 5637	[127]
6	SPC, Chol		Curcumin, TAT-PEG-SN38	Hydrodynamic flow-focusing	171	−5.94	0.124	88.4 (Curcumin)	A549	[131]
7	DOTAP, DSPE-PEG2000, DSPC, Chol		Copper complexed Chlorin e6 (Cu-Ce6)	Serpentine mixer	50–100	−15 to 35	0.1–0.6	–	HeLa	[128]
8	DPPC, DSPC, chol, DSPE-MPEG2000	Indocyanine green	Ansamitocin P-3	Microfluidic swirl mixer	125	−8.3	0.04	51 (Indocyanine green)/81 (Ansamitocin P-3)	MCF-7	[132]
9	JK-102-CA, Chol, DSPC, PEG2000-DMG	Nicotinamide mononucleotide	Honokiol	Microfluidic membrane emulsification device	164	–	>0.2	28.4 (Nicotinamide mononucleotide)/99.2 (Honokiol)	HCT-116	[133]

Abbreviations: 17-AAG, 17-allylamino-17-demethoxygeldanamycin; Chol, cholesterol; DMG, dimyristoyl glycerol; DOPC, dioleoylphosphatidylcholine; DOTAP, 1,2-dioleoyl-3-trimethylammonium-propane; DPPC, dipalmitoylphosphatidycholine; DSPC, distearoylphosphatidylcholine; EE, encapsulation efficiency; FA, folic acid; HDA, hexadecylamine; MPEG, methoxy poly(ethylene glycol); PDI, polydispersity index; PEG, polyethylene glycol; POPC, palmitoyloleoylphosphatidylcholine; SPC, soy phosphatidylcholine; TAT-PEG-SN38, an amphiphilic camptothecin prodrug (TAT-PEG-SN38) linked to PEG and cell-penetrating peptide (TAT); Z.P., zeta potential.

## Data Availability

Not applicable.

## References

[1] Kadam U.T., Roberts I., White S., Bednall R., Khunti K., Nilsson P.M., Lawson C.A. (2019). Conceptualizing multiple drug use in patients with comorbidity and multimorbidity: Proposal for standard definitions beyond the term polypharmacy. J. Clin. Epidemiol..

[2] Mortezaee K., Majidpoor J. (2022). Checkpoint inhibitor/interleukin-based combination therapy of cancer. Cancer Med..

[3] Wang X., Li J., Chen R., Li T., Chen M. (2023). Active Ingredients from Chinese Medicine for Combination Cancer Therapy. Int. J. Biol. Sci..

[4] Blumer V., Vaduganathan M. (2022). A rationale for dedicated trials of combination therapy in heart failure. Eur. Heart J. Suppl..

[5] Bauersachs J. (2021). Heart failure drug treatment: The fantastic four. Eur. Heart J..

[6] Xie X., Wu C., Hao Y., Wang T., Yang Y., Cai P., Zhang Y., Huang J., Deng K., Yan D. (2023). Benefits and risks of drug combination therapy for diabetes mellitus and its complications: A comprehensive review. Front. Endocrinol..

[7] Qiu T., Yan D. (2024). Editorial: Benefits and risks of drug combination therapy for chronic metabolic diseases. Front. Endocrinol..

[8] Huuskes B.M., Wise A.F., Cox A.J., Lim E.X., Payne N.L., Kelly D.J., Samuel C.S., Ricardo S.D. (2015). Combination therapy of mesenchymal stem cells and serelaxin effectively attenuates renal fibrosis in obstructive nephropathy. FASEB J..

[9] Kabir M.T., Uddin M.S., Mamun A.A., Jeandet P., Aleya L., Mansouri R.A., Ashraf G.M., Mathew B., Bin-Jumah M.N., Abdel-Daim M.M. (2020). Combination Drug Therapy for the Management of Alzheimer’s Disease. Int. J. Mol. Sci..

[10] Lagerberg T., Sjölander A., Gibbons R.D., Quinn P.D., D’Onofrio B.M., Hellner C., Lichtenstein P., Fazel S., Chang Z. (2022). Use of central nervous system drugs in combination with selective serotonin reuptake inhibitor treatment: A Bayesian screening study for risk of suicidal behavior. Front. Psychiatry.

[11] Chrastina M., Poništ S., Tóth J., Czigle S., Pašková Ľ., Vyletelová V., Švík K., Bauerová K. (2022). Combination Therapy of Carnosic Acid and Methotrexate Effectively Suppressed the Inflammatory Markers and Oxidative Stress in Experimental Arthritis. Molecules.

[12] Huang R., Zhang C., Bu Y., Li Z., Zheng X., Qiu S., Machuki J.O.a., Zhang L., Yang Y., Guo K. (2021). A multifunctional nano-therapeutic platform based on octahedral yolk-shell Au NR@CuS: Photothermal/photodynamic and targeted drug delivery tri-combined therapy for rheumatoid arthritis. Biomaterials.

[13] de Souza G.E., Bueno R.V., de Souza J.O., Zanini C.L., Cruz F.C., Oliva G., Guido R.V.C., Aguiar A.C.C. (2019). Antiplasmodial profile of selected compounds from Malaria Box: In vitro evaluation, speed of action and drug combination studies. Malar. J..

[14] Matsuda K., Maeda K. (2024). HIV Reservoirs and Treatment Strategies toward Curing HIV Infection. Int. J. Mol. Sci..

[15] Nath B.J., Sadri K., Sarmah H.K., Hosseini K. (2024). An optimal combination of antiretroviral treatment and immunotherapy for controlling HIV infection. Math. Comput. Simul..

[16] Dartois V., Dick T. (2024). Therapeutic developments for tuberculosis and nontuberculous mycobacterial lung disease. Nat. Rev. Drug Discov..

[17] Dawson R., Diacon A.H., Takuva S., Liu Y., Zheng B., Karwe V., Hafkin J. (2024). Quabodepistat in combination with delamanid and bedaquiline in participants with drug-susceptible pulmonary tuberculosis: Protocol for a multicenter, phase 2b/c, open-label, randomized, dose-finding trial to evaluate safety and efficacy. Trials.

[18] Smith C.S., Aerts A., Saunderson P., Kawuma J., Kita E., Virmond M. (2017). Multidrug therapy for leprosy: A game changer on the path to elimination. Lancet Infect. Dis..

[19] Sun W., Sanderson P.E., Zheng W. (2016). Drug combination therapy increases successful drug repositioning. Drug Discov. Today.

[20] Brown J.S., Amend S.R., Austin R.H., Gatenby R.A., Hammarlund E.U., Pienta K.J. (2023). Updating the Definition of Cancer. Mol. Cancer Res..

[21] Anand U., Dey A., Chandel A.K.S., Sanyal R., Mishra A., Pandey D.K., De Falco V., Upadhyay A., Kandimalla R., Chaudhary A. (2023). Cancer chemotherapy and beyond: Current status, drug candidates, associated risks and progress in targeted therapeutics. Genes Dis..

[22] Boshuizen J., Peeper D.S. (2020). Rational Cancer Treatment Combinations: An Urgent Clinical Need. Mol. Cell.

[23] National Cancer Institute Drugs Approved for Different Types of Cancer. https://www.cancer.gov/about-cancer/treatment/drugs/cancer-type.

[24] Duarte D., Vale N. (2022). Evaluation of synergism in drug combinations and reference models for future orientations in oncology. Curr. Res. Pharmacol. Drug Discov..

[25] Dentro S.C., Leshchiner I., Haase K., Tarabichi M., Wintersinger J., Deshwar A.G., Yu K., Rubanova Y., Macintyre G., Demeulemeester J. (2021). Characterizing genetic intra-tumor heterogeneity across 2,658 human cancer genomes. Cell.

[26] Zhang A., Miao K., Sun H., Deng C.X. (2022). Tumor heterogeneity reshapes the tumor microenvironment to influence drug resistance. Int. J. Biol. Sci..

[27] Jaaks P., Coker E.A., Vis D.J., Edwards O., Carpenter E.F., Leto S.M., Dwane L., Sassi F., Lightfoot H., Barthorpe S. (2022). Effective drug combinations in breast, colon and pancreatic cancer cells. Nature.

[28] Peng Y., Wang Y., Zhou C., Mei W., Zeng C. (2022). PI3K/Akt/mTOR Pathway and Its Role in Cancer Therapeutics: Are We Making Headway?. Front. Oncol..

[29] Deng J., Bai X., Feng X., Ni J., Beretov J., Graham P., Li Y. (2019). Inhibition of PI3K/Akt/mTOR signaling pathway alleviates ovarian cancer chemoresistance through reversing epithelial-mesenchymal transition and decreasing cancer stem cell marker expression. BMC Cancer.

[30] Jin M.Z., Jin W.L. (2020). The updated landscape of tumor microenvironment and drug repurposing. Signal Transduct. Target. Ther..

[31] Patel S.A., Nilsson M.B., Le X., Cascone T., Jain R.K., Heymach J.V. (2023). Molecular Mechanisms and Future Implications of VEGF/VEGFR in Cancer Therapy. Clin. Cancer Res..

[32] Hanahan D., Weinberg R.A. (2011). Hallmarks of cancer: The next generation. Cell.

[33] Zhao H., Wu L., Yan G., Chen Y., Zhou M., Wu Y., Li Y. (2021). Inflammation and tumor progression: Signaling pathways and targeted intervention. Signal Transduct. Target. Ther..

[34] Liu X., Yin L., Shen S., Hou Y. (2023). Inflammation and cancer: Paradoxical roles in tumorigenesis and implications in immunotherapies. Genes Dis..

[35] Gómez-Valenzuela F., Escobar E., Pérez-Tomás R., Montecinos V.P. (2021). The Inflammatory Profile of the Tumor Microenvironment, Orchestrated by Cyclooxygenase-2, Promotes Epithelial-Mesenchymal Transition. Front. Oncol..

[36] Kolawole O.R., Kashfi K. (2022). NSAIDs and Cancer Resolution: New Paradigms beyond Cyclooxygenase. Int. J. Mol. Sci..

[37] Rodrigues P., Bangali H., Hammoud A., Mustafa Y.F., Al-Hetty H.R.A.K., Alkhafaji A.T., Deorari M.M., Al-Taee M.M., Zabibah R.S., Alsalamy A. (2024). COX 2-inhibitors; a thorough and updated survey into combinational therapies in cancers. Med. Oncol..

[38] Changou C.A., Shiah H.S., Chen L.T., Liu S., Luh F., Liu S.H., Cheng Y.C., Yen Y. (2020). A Phase II Clinical Trial on the Combination Therapy of PHY906 Plus Capecitabine in Hepatocellular Carcinoma. Oncologist.

[39] Marcucci F., Berenson R., Corti A. (2013). Improving drug uptake and penetration into tumors: Current and forthcoming opportunities. Front. Oncol..

[40] Alimoradi H., Greish K., Barzegar-Fallah A., Alshaibani L., Pittalà V. (2018). Nitric oxide-releasing nanoparticles improve doxorubicin anticancer activity. Int. J. Nanomed..

[41] Hingorani S.R., Harris W.P., Beck J.T., Berdov B.A., Wagner S.A., Pshevlotsky E.M., Tjulandin S.A., Gladkov O.A., Holcombe R.F., Korn R. (2016). Phase Ib Study of PEGylated Recombinant Human Hyaluronidase and Gemcitabine in Patients with Advanced Pancreatic Cancer. Clin. Cancer Res..

[42] Beyer I., van Rensburg R., Strauss R., Li Z., Wang H., Persson J., Yumul R., Feng Q., Song H., Bartek J. (2011). Epithelial junction opener JO-1 improves monoclonal antibody therapy of cancer. Cancer Res..

[43] Ramirez-Velez I., Belardi B. (2023). Storming the gate: New approaches for targeting the dynamic tight junction for improved drug delivery. Adv. Drug Deliv. Rev..

[44] Li P., Zhong D., Gong P.Y. (2019). Synergistic effect of paclitaxel and verapamil to overcome multi-drug resistance in breast cancer cells. Biochem. Biophys. Res. Commun..

[45] Ocaña-Arakachi K., Martínez-Herculano J., Jurado R., Llaguno-Munive M., Garcia-Lopez P. (2023). Pharmacokinetics and Anti-Tumor Efficacy of PEGylated Liposomes Co-Loaded with Cisplatin and Mifepristone. Pharmaceuticals.

[46] Yan V.C., Butterfield H.E., Poral A.H., Yan M.J., Yang K.L., Pham C.D., Muller F.L. (2020). Why Great Mitotic Inhibitors Make Poor Cancer Drugs. Trends Cancer.

[47] Alfarouk K.O., Stock C.-M., Taylor S., Walsh M., Muddathir A.K., Verduzco D., Bashir A.H.H., Mohammed O.Y., Elhassan G.O., Harguindey S. (2015). Resistance to cancer chemotherapy: Failure in drug response from ADME to P-gp. Cancer Cell Int..

[48] Tolcher A.W., Mayer L.D. (2018). Improving combination cancer therapy: The CombiPlex^®^ development platform. Future Oncol..

[49] Liboiron B.D., Mayer L.D. (2014). Nanoscale particulate systems for multidrug delivery: Towards improved combination chemotherapy. Ther. Deliv..

[50] Zhu L., Jiang M., Wang H., Sun H., Zhu J., Zhao W., Fang Q., Yu J., Chen P., Wu S. (2021). A narrative review of tumor heterogeneity and challenges to tumor drug therapy. Ann. Transl. Med..

[51] Sultana A., Zare M., Thomas V., Kumar T.S.S., Ramakrishna S. (2022). Nano-based drug delivery systems: Conventional drug delivery routes, recent developments and future prospects. Med. Drug Discov..

[52] Torchilin V.P. (2005). Recent advances with liposomes as pharmaceutical carriers. Nat. Rev. Drug Discov..

[53] Nsairat H., Khater D., Sayed U., Odeh F., Al Bawab A., Alshaer W. (2022). Liposomes: Structure, composition, types, and clinical applications. Heliyon.

[54] Chen J., Hu S., Sun M., Shi J., Zhang H., Yu H., Yang Z. (2024). Recent advances and clinical translation of liposomal delivery systems in cancer therapy. Eur. J. Pharm. Sci..

[55] Abbasi H., Kouchak M., Mirveis Z., Hajipour F., Khodarahmi M., Rahbar N., Handali S. (2023). What We Need to Know about Liposomes as Drug Nanocarriers: An Updated Review. Adv. Pharm. Bull..

[56] Yatvin M.B., Lelkes P.I. (1982). Clinical prospects for liposomes. Med. Phys..

[57] Sanati M., Afshari A.R., Ahmadi S.S., Kesharwani P., Sahebkar A. (2024). Advances in liposome-based delivery of RNA therapeutics for cancer treatment. Prog. Mol. Biol. Transl. Sci..

[58] Miatmoko A., Octavia R.T., Araki T., Annoura T., Sari R. (2024). Advancing liposome technology for innovative strategies against malaria. Saudi Pharm. J..

[59] Ashfaq R., Rasul A., Asghar S., Kovács A., Berkó S., Budai-Szűcs M. (2023). Lipid Nanoparticles: An Effective Tool to Improve the Bioavailability of Nutraceuticals. Int. J. Mol. Sci..

[60] Patra J.K., Das G., Fraceto L.F., Campos E.V.R., Rodriguez-Torres M.d.P., Acosta-Torres L.S., Diaz-Torres L.A., Grillo R., Swamy M.K., Sharma S. (2018). Nano based drug delivery systems: Recent developments and future prospects. J. Nanobiotechnol..

[61] Lombardo D., Kiselev M.A. (2022). Methods of Liposomes Preparation: Formation and Control Factors of Versatile Nanocarriers for Biomedical and Nanomedicine Application. Pharmaceutics.

[62] Maja L., Željko K., Mateja P. (2020). Sustainable technologies for liposome preparation. J. Supercrit. Fluids.

[63] Wang S., Chen Y., Guo J., Huang Q. (2023). Liposomes for Tumor Targeted Therapy: A Review. Int. J. Mol. Sci..

[64] Liu Z., Fontana F., Python A., Hirvonen J.T., Santos H.A. (2020). Microfluidics for Production of Particles: Mechanism, Methodology, and Applications. Small.

[65] Kotouček J., Hubatka F., Mašek J., Kulich P., Velínská K., Bezděková J., Fojtíková M., Bartheldyová E., Tomečková A., Stráská J. (2020). Preparation of nanoliposomes by microfluidic mixing in herring-bone channel and the role of membrane fluidity in liposomes formation. Sci. Rep..

[66] Gbian D.L., Omri A. (2022). Lipid-Based Drug Delivery Systems for Diseases Managements. Biomedicines.

[67] Sebastian V. (2022). Toward continuous production of high-quality nanomaterials using microfluidics: Nanoengineering the shape, structure and chemical composition. Nanoscale.

[68] Naghib S.M., Mohammad-Jafari K. (2024). Microfluidics-mediated Liposomal Nanoparticles for Cancer Therapy: Recent Developments on Advanced Devices and Technologies. Curr. Top. Med. Chem..

[69] Webb C., Forbes N., Roces C.B., Anderluzzi G., Lou G., Abraham S., Ingalls L., Marshall K., Leaver T.J., Watts J.A. (2020). Using microfluidics for scalable manufacturing of nanomedicines from bench to GMP: A case study using protein-loaded liposomes. Int. J. Pharm..

[70] Akar S., Fardindoost S., Hoorfar M. (2024). High throughput microfluidics-based synthesis of PEGylated liposomes for precise size control and efficient drug encapsulation. Colloids Surf. B Biointerfaces.

[71] Han J.Y., La Fiandra J.N., DeVoe D.L. (2022). Microfluidic vortex focusing for high throughput synthesis of size-tunable liposomes. Nat. Commun..

[72] Forbes N., Hussain M.T., Briuglia M.L., Edwards D.P., Horst J.H.t., Szita N., Perrie Y. (2019). Rapid and scale-independent microfluidic manufacture of liposomes entrapping protein incorporating in-line purification and at-line size monitoring. Int. J. Pharm..

[73] Zizzari A., Bianco M., Carbone L., Perrone E., Amato F., Maruccio G., Rendina F., Arima V. (2017). Continuous-Flow Production of Injectable Liposomes via a Microfluidic Approach. Materials.

[74] Michelon M., Oliveira D.R.B., de Figueiredo Furtado G., Gaziola de la Torre L., Cunha R.L. (2017). High-throughput continuous production of liposomes using hydrodynamic flow-focusing microfluidic devices. Colloids Surf. B Biointerfaces.

[75] Ota A., Mochizuki A., Sou K., Takeoka S. (2023). Evaluation of a static mixer as a new microfluidic method for liposome formulation. Front. Bioeng. Biotechnol..

[76] Bing L., Hao M., Zhenbo T. (2024). Preparation of lipid nanoparticles by micro-mixer Process simulation and experimental study. BIO Web Conf..

[77] Ceccato B.T., Vianna S.S.V., de la Torre L.G. (2024). Numerical and experimental investigation of chaotic advection and diffusion mixing effects in 3D multihelical microfluidics for liposome synthesis. Chem. Eng. Sci..

[78] Shah V.M., Nguyen D.X., Patel P., Cote B., Al-Fatease A., Pham Y., Huynh M.G., Woo Y., Alani A.W. (2019). Liposomes produced by microfluidics and extrusion: A comparison for scale-up purposes. Nanomedicine.

[79] López R.R., Ocampo I., Font de Rubinat P.G., Sánchez L.M., Alazzam A., Tsering T., Bergeron K.F., Camacho-Léon S., Burnier J.V., Mounier C. (2021). Parametric Study of the Factors Influencing Liposome Physicochemical Characteristics in a Periodic Disturbance Mixer. Langmuir.

[80] Weaver E., Mathew E., Caldwell J., Hooker A., Uddin S., Lamprou D.A. (2023). The manufacturing of 3D-printed microfluidic chips to analyse the effect upon particle size during the synthesis of lipid nanoparticles. J. Pharm. Pharmacol..

[81] Liu P., Chen G., Zhang J. (2022). A Review of Liposomes as a Drug Delivery System: Current Status of Approved Products, Regulatory Environments, and Future Perspectives. Molecules.

[82] Briuglia M.L., Rotella C., McFarlane A., Lamprou D.A. (2015). Influence of cholesterol on liposome stability and on in vitro drug release. Drug Deliv. Transl. Res..

[83] Choi S., Kang B., Yang E., Kim K., Kwak M.K., Chang P.S., Jung H.S. (2023). Precise control of liposome size using characteristic time depends on solvent type and membrane properties. Sci. Rep..

[84] Maeki M., Kimura N., Okada Y., Shimizu K., Shibata K., Miyazaki Y., Ishida A., Yonezawa K., Shimizu N., Shinoda W. (2024). Understanding the effects of ethanol on the liposome bilayer structure using microfluidic-based time-resolved small-angle X-ray scattering and molecular dynamics simulations. Nanoscale Adv..

[85] Xu X., Khan M.A., Burgess D.J. (2012). Predicting hydrophilic drug encapsulation inside unilamellar liposomes. Int. J. Pharm..

[86] Guimarães D., Cavaco-Paulo A., Nogueira E. (2021). Design of liposomes as drug delivery system for therapeutic applications. Int. J. Pharm..

[87] Saorin A., Saorin G., Duzagac F., Parisse P., Cao N., Corona G., Cavarzerani E., Rizzolio F. (2024). Microfluidic production of amiodarone loaded nanoparticles and application in drug repositioning in ovarian cancer. Sci. Rep..

[88] Li T., Cipolla D., Rades T., Boyd B.J. (2018). Drug nanocrystallisation within liposomes. J. Control. Release.

[89] Pisani S., Di Martino D., Cerri S., Genta I., Dorati R., Bertino G., Benazzo M., Conti B. (2023). Investigation and Comparison of Active and Passive Encapsulation Methods for Loading Proteins into Liposomes. Int. J. Mol. Sci..

[90] Nam J.H., Kim S.Y., Seong H. (2018). Investigation on Physicochemical Characteristics of a Nanoliposome-Based System for Dual Drug Delivery. Nanoscale Res. Lett..

[91] Jaradat E., Meziane A., Lamprou D.A. (2024). Conventional vs PEGylated loaded liposomal formulations by microfluidics for delivering hydrophilic chemotherapy. Int. J. Pharm..

[92] Adepu S., Ramakrishna S. (2021). Controlled Drug Delivery Systems: Current Status and Future Directions. Molecules.

[93] Đorđević S., Gonzalez M.M., Conejos-Sánchez I., Carreira B., Pozzi S., Acúrcio R.C., Satchi-Fainaro R., Florindo H.F., Vicent M.J. (2022). Current hurdles to the translation of nanomedicines from bench to the clinic. Drug Deliv. Transl. Res..

[94] Zhang R.X., Wong H.L., Xue H.Y., Eoh J.Y., Wu X.Y. (2016). Nanomedicine of synergistic drug combinations for cancer therapy-Strategies and perspectives. J. Control. Release.

[95] Wu M., Lin X., Tan X., Li J., Wei Z., Zhang D., Zheng Y., Zheng A.X., Zhao B., Zeng Y. (2018). Photoresponsive Nanovehicle for Two Independent Wavelength Light-Triggered Sequential Release of P-gp shRNA and Doxorubicin To Optimize and Enhance Synergistic Therapy of Multidrug-Resistant Cancer. ACS Appl. Mater. Interfaces.

[96] Andra V., Pammi S.V.N., Bhatraju L., Ruddaraju L.K. (2022). A Comprehensive Review on Novel Liposomal Methodologies, Commercial Formulations, Clinical Trials and Patents. Bionanoscience.

[97] Nounou M.M., El-Khordagui L.K., Khalafallah N.A., Khalil S.A. (2006). In vitro release of hydrophilic and hydrophobic drugs from liposomal dispersions and gels. Acta Pharm..

[98] Song F., Yang G., Wang Y., Tian S. (2022). Effect of phospholipids on membrane characteristics and storage stability of liposomes. Innov. Food Sci. Emerg. Technol..

[99] Pamunuwa G., Karunaratne V., Karunaratne D.N. (2016). Effect of Lipid Composition on In Vitro Release and Skin Deposition of Curcumin Encapsulated Liposomes. J. Nanomater..

[100] Sheikholeslami B., Lam N.W., Dua K., Haghi M. (2022). Exploring the impact of physicochemical properties of liposomal formulations on their in vivo fate. Life Sci..

[101] Liu Y., Tamam H., Yeo Y. (2018). Mixed Liposome Approach for Ratiometric and Sequential Delivery of Paclitaxel and Gemcitabine. AAPS PharmSciTech.

[102] Tang T., Gong Y., Gao Y., Pang X., Liu S., Xia Y., Liu D., Zhu L., Fan Q., Sun X. (2023). A pH-responsive liposomal nanoplatform for co-delivery of a Pt(IV) prodrug and cinnamaldehyde for effective tumor therapy. Front. Bioeng. Biotechnol..

[103] Fouladi F., Steffen K.J., Mallik S. (2017). Enzyme-Responsive Liposomes for the Delivery of Anticancer Drugs. Bioconjug. Chem..

[104] Tang L., Wang Y.J., Wang Y.Y., Li S.T., Kong L., Li X.T., Ma L.L., Liu X.X. (2024). Construction of ROS-Responsive Hyaluronic Acid Modified Paclitaxel and Diosgenin Liposomes and Study on Synergistic Enhancement of Anti-Ovarian Cancer Efficacy. Int. J. Nanomed..

[105] Fahmy S.A., Preis E., Dayyih A.A., Alawak M., El-Said Azzazy H.M., Bakowsky U., Shoeib T. (2022). Thermosensitive Liposomes Encapsulating Nedaplatin and Picoplatin Demonstrate Enhanced Cytotoxicity against Breast Cancer Cells. ACS Omega.

[106] Agiba A.M., Arreola-Ramírez J.L., Carbajal V., Segura-Medina P. (2024). Light-Responsive and Dual-Targeting Liposomes: From Mechanisms to Targeting Strategies. Molecules.

[107] Wang C., Zhang R., He J., Yu L., Li X., Zhang J., Li S., Zhang C., Kagan J.C., Karp J.M. (2023). Ultrasound-responsive low-dose doxorubicin liposomes trigger mitochondrial DNA release and activate cGAS-STING-mediated antitumour immunity. Nat. Commun..

[108] Pilkington C.P., Gispert I., Chui S.Y., Seddon J.M., Elani Y. (2024). Engineering a nanoscale liposome-in-liposome for in situ biochemical synthesis and multi-stage release. Nat. Chem..

[109] Sainaga Jyothi V.G.S., Bulusu R., Venkata Krishna Rao B., Pranothi M., Banda S., Kumar Bolla P., Kommineni N. (2022). Stability characterization for pharmaceutical liposome product development with focus on regulatory considerations: An update. Int. J. Pharm..

[110] Xu X., Tian F., Pan Y., Zhang T., Deng L., Jiang H., Han J., Liu J., Zhao Y., Liu W. (2025). Emerging mechanistic insights into liposomal stability: Full process management from production and storage to food application. Chem. Eng. J..

[111] Lehman S.E., Benkstein K.D., Cleveland T.E.I.V., Anderson K.W., Carrier M.J., Vreeland W.N. (2023). Particle Metrology Approach to Understanding How Storage Conditions Affect Long-Term Liposome Stability. Langmuir.

[112] Musakhanian J., Rodier J.D., Dave M. (2022). Oxidative Stability in Lipid Formulations: A Review of the Mechanisms, Drivers, and Inhibitors of Oxidation. AAPS PharmSciTech.

[113] Oude Blenke E., Örnskov E., Schöneich C., Nilsson G.A., Volkin D.B., Mastrobattista E., Almarsson Ö., Crommelin D.J.A. (2023). The Storage and In-Use Stability of mRNA Vaccines and Therapeutics: Not A Cold Case. J. Pharm. Sci..

[114] Crommelin D.J.A., van Hoogevest P., Storm G. (2020). The role of liposomes in clinical nanomedicine development. What now? Now what?. J. Control. Release.

[115] Andreana I., Bincoletto V., Manzoli M., Rodà F., Giarraputo V., Milla P., Arpicco S., Stella B. (2023). Freeze Drying of Polymer Nanoparticles and Liposomes Exploiting Different Saccharide-Based Approaches. Materials.

[116] Pasarin D., Ghizdareanu A.I., Enascuta C.E., Matei C.B., Bilbie C., Paraschiv-Palada L., Veres P.A. (2023). Coating Materials to Increase the Stability of Liposomes. Polymers.

[117] Sydykov B., Oldenhof H., Sieme H., Wolkers W.F. (2018). Storage stability of liposomes stored at elevated subzero temperatures in DMSO/sucrose mixtures. PLoS ONE.

[118] Kong F., Zhang X., Zhang H., Qu X., Chen D., Servos M., Mäkilä E., Salonen J., Santos H.A., Hai M. (2015). Inhibition of Multidrug Resistance of Cancer Cells by Co-Delivery of DNA Nanostructures and Drugs Using Porous Silicon Nanoparticles@Giant Liposomes. Adv. Funct. Mater..

[119] Gkionis L., Campbell R.A., Aojula H., Harris L.K., Tirella A. (2020). Manufacturing drug co-loaded liposomal formulations targeting breast cancer: Influence of preparative method on liposomes characteristics and in vitro toxicity. Int. J. Pharm..

[120] Wong S.C., Kamarudin M.N.A., Naidu R. (2021). Anticancer Mechanism of Curcumin on Human Glioblastoma. Nutrients.

[121] Ojo O.A., Adeyemo T.R., Rotimi D., Batiha G.E., Mostafa-Hedeab G., Iyobhebhe M.E., Elebiyo T.C., Atunwa B., Ojo A.B., Lima C.M.G. (2022). Anticancer Properties of Curcumin Against Colorectal Cancer: A Review. Front. Oncol..

[122] Dytrych P., Kejík Z., Hajduch J., Kaplánek R., Veselá K., Kučnirová K., Skaličková M., Venhauerová A., Hoskovec D., Martásek P. (2023). Therapeutic potential and limitations of curcumin as antimetastatic agent. Biomed. Pharmacother..

[123] Tabanelli R., Brogi S., Calderone V. (2021). Improving Curcumin Bioavailability: Current Strategies and Future Perspectives. Pharmaceutics.

[124] Hong S.-C., Park K.-M., Hong C.R., Kim J.-C., Yang S.-H., Yu H.-S., Paik H.-D., Pan C.-H., Chang P.-S. (2020). Microfluidic assembly of liposomes dual-loaded with catechin and curcumin for enhancing bioavailability. Colloids Surf. Physicochem. Eng. Asp..

[125] Gong L., Zhang Y., Liu C., Zhang M., Han S. (2021). Application of Radiosensitizers in Cancer Radiotherapy. Int. J. Nanomed..

[126] Charest G., Tippayamontri T., Shi M., Wehbe M., Anantha M., Bally M., Sanche L. (2020). Concomitant Chemoradiation Therapy with Gold Nanoparticles and Platinum Drugs Co-Encapsulated in Liposomes. Int. J. Mol. Sci..

[127] Lv S., Jing R., Liu X., Shi H., Shi Y., Wang X., Zhao X., Cao K., Lv Z. (2021). One-Step Microfluidic Fabrication of Multi-Responsive Liposomes for Targeted Delivery of Doxorubicin Synergism with Photothermal Effect. Int. J. Nanomed..

[128] Li M., Liu C., Yin J., Liu G., Chen D. (2022). Single-Step Synthesis of Highly Tunable Multifunctional Nanoliposomes for Synergistic Cancer Therapy. ACS Appl. Mater. Interfaces.

[129] Donkor M., Jones H.P. (2021). The Proposition of the Pulmonary Route as an Attractive Drug Delivery Approach of Nano-Based Immune Therapies and Cancer Vaccines to Treat Lung Tumors. Front. Nanotechnol..

[130] Rong A., Han Z., Wang T., Zhu M., Zhou M., Sun X. (2024). Pulmonary delivery of nano-particles for lung cancer diagnosis and therapy: Recent advances and future prospects. WIREs Nanomed. Nanobiotechnol..

[131] Gao C., Zhang L., Xu M., Luo Y., Wang B., Kuang M., Liu X., Sun M., Guo Y., Teng L. (2022). Pulmonary delivery of liposomes co-loaded with SN38 prodrug and curcumin for the treatment of lung cancer. Eur. J. Pharm. Biopharm..

[132] Jin Y., Tomeh M.A., Zhang P., Su M., Zhao X., Cai Z. (2023). Microfluidic fabrication of photo-responsive Ansamitocin P-3 loaded liposomes for the treatment of breast cancer. Nanoscale.

[133] Zhang L., Zhu H., Ye P., Zhu L., Ren Y., Lei J. (2024). Controlled production of liposomes with novel microfluidic membrane emulsification for application of entrapping hydrophilic and lipophilic drugs. J. Ind. Eng. Chem..

[134] Wu J. (2021). The Enhanced Permeability and Retention (EPR) Effect: The Significance of the Concept and Methods to Enhance Its Application. J. Pers. Med..

[135] Ran R., Middelberg A.P.J., Zhao C.X. (2016). Microfluidic synthesis of multifunctional liposomes for tumour targeting. Colloids Surf. B Biointerfaces.

[136] Dacos M., Immordino B., Diroff E., Sicard G., Kosta A., Rodallec A., Giacometti S., Ciccolini J., Fanciullino R. (2024). Pegylated liposome encapsulating docetaxel using microfluidic mixing technique: Process optimization and results in breast cancer models. Int. J. Pharm..

[137] Arduino I., Di Fonte R., Sommonte F., Lopedota A.A., Porcelli L., Li J., Serrati S., Bártolo R., Santos H.A., Iacobazzi R.M. (2024). Fabrication of Biomimetic Hybrid Liposomes via Microfluidic Technology: Homotypic Targeting and Antitumor Efficacy Studies in Glioma Cells. Int. J. Nanomed..

[138] Shan H., Sun X., Liu X., Sun Q., He Y., Chen Z., Lin Q., Jiang Z., Chen X., Chen Z. (2023). One-Step Formation of Targeted Liposomes in a Versatile Microfluidic Mixing Device. Small.

[139] Thompson G.R., Sani G.M., Donnelley M.A., Figueroa J.K., Ciuffetelli R., Trigg K., Arredondo J., Koff A., Nearing M., Loque I.C. (2025). Liposomal amphotericin B and complement activation-related pseudoallergy (CARPA). Antimicrob. Agents Chemother..

[140] Gordon S., Gregoriadis G., McCormack B. (1998). The Mononuclear Phagocyte System: Features Relevant to Interactions with Liposomes. Targeting of Drugs 6: Strategies for Stealth Therapeutic Systems.

[141] Belyaev I.B., Mirkasymov A.B., Rodionov V.I., Babkova J.S., Nikitin P.I., Deyev S.M., Zelepukin I.V. (2024). MPS blockade with liposomes controls pharmacokinetics of nanoparticles in a size-dependent manner. Biomed. Mater..

[142] Taher M., Susanti D., Haris M.S., Rushdan A.A., Widodo R.T., Syukri Y., Khotib J. (2023). PEGylated liposomes enhance the effect of cytotoxic drug: A review. Heliyon.

[143] Wang H., Wang Y., Yuan C., Xu X., Zhou W., Huang Y., Lu H., Zheng Y., Luo G., Shang J. (2023). Polyethylene glycol (PEG)-associated immune responses triggered by clinically relevant lipid nanoparticles in rats. NPJ Vaccines.

[144] Zhang Z., Sun H., Giannino J., Wu Y., Cheng C. (2024). Biodegradable zwitterionic polymers as PEG alternatives for drug delivery. J. Polym. Sci..

[145] Yao X., Qi C., Sun C., Huo F., Jiang X. (2023). Poly(ethylene glycol) alternatives in biomedical applications. Nano Today.

[146] Kurzrock R., Lin C.C., Wu T.C., Hobbs B.P., Pestana R.M., Hong D.S. (2021). Moving Beyond 3+3: The Future of Clinical Trial Design. Am. Soc. Clin. Oncol. Educ. Book.

[147] Ha H., Lee H.Y., Kim J.H., Kim D.Y., An H.J., Bae S., Park H.-S., Kang J.H. (2024). Precision Oncology Clinical Trials: A Systematic Review of Phase II Clinical Trials with Biomarker-Driven, Adaptive Design. Cancer Res. Treat..

[148] Peyrin-Biroulet L., Lopez A., Sandborn W. (2016). Head-to-head Compsarative Studies: Challenges and Opportunities?. J. Crohn's Colitis.

[149] Dagenais S., Russo L., Madsen A., Webster J., Becnel L. (2022). Use of Real-World Evidence to Drive Drug Development Strategy and Inform Clinical Trial Design. Clin. Pharmacol. Ther..

[150] Crestan D., Trojniak M.P., Francescon S., Fornasier G., Baldo P. (2020). Pharmacovigilance of anti-cancer medicines: Opportunities and challenges. Expert Opin. Drug Saf..

[151] Mayer L.D., Tardi P., Louie A.C. (2019). CPX-351: A nanoscale liposomal co-formulation of daunorubicin and cytarabine with unique biodistribution and tumor cell uptake properties. Int. J. Nanomed..

[152] Blair H.A. (2018). Daunorubicin/Cytarabine Liposome: A Review in Acute Myeloid Leukaemia. Drugs.

[153] Zhang H., Yang J., Sun R., Han S., Yang Z., Teng L. (2023). Microfluidics for nano-drug delivery systems: From fundamentals to industrialization. Acta Pharm. Sin. B.

[154] Sealy A. (2021). Manufacturing Moonshot: How Pfizer Makes Its Millions of Covid-19 Vaccine Doses.

